# Nuclear phosphorylated Dicer processes double-stranded RNA in response to DNA damage

**DOI:** 10.1083/jcb.201612131

**Published:** 2017-08-07

**Authors:** Kaspar Burger, Margarita Schlackow, Martin Potts, Svenja Hester, Shabaz Mohammed, Monika Gullerova

**Affiliations:** 1 Sir William Dunn School of Pathology, University of Oxford, Oxford, UK; 2 Department of Biochemistry, University of Oxford, Oxford, UK; 3 Cambridge Institute for Medical Research, University of Cambridge, Cambridge, UK

## Abstract

The endoribonuclease Dicer is a key component of the human RNA interference pathway and is known for its role in cytoplasmic microRNA production. Recent findings suggest that noncanonical Dicer generates small noncoding RNA to mediate the DNA damage response (DDR). Here, we show that human Dicer is phosphorylated in the platform–Piwi/Argonaute/Zwille–connector helix cassette (S1016) upon induction of DNA damage. Phosphorylated Dicer (p-Dicer) accumulates in the nucleus and is recruited to DNA double-strand breaks. We further demonstrate that turnover of damage-induced nuclear, double-stranded (ds) RNA requires additional phosphorylation of carboxy-terminal Dicer residues (S1728 and S1852). DNA damage-induced nuclear Dicer accumulation is conserved in mammals. Dicer depletion causes endogenous DNA damage and delays the DDR by impaired recruitment of repair factors MDC1 and 53BP1. Collectively, we place Dicer within the context of the DDR by demonstrating a DNA damage-inducible phosphoswitch that causes localized processing of nuclear dsRNA by p-Dicer to promote DNA repair.

## Abstract (precis)

Recent work implicated human Dicer in the DNA damage response. Burger et al. show that DNA damage induces phosphorylation of Dicer and promotes DNA repair in the nucleus.

## Introduction

The endoribonuclease Dicer is a key component of the RNAi pathway. Dicer processing generates 20–25-nt-long miRNA from a stem-loop precursor miRNA (Chendrimada et al., 2005; Haase et al., 2005). Mature miRNA are loaded onto the Argonaute-containing, RNA-induced silencing complex to target complementary mRNA for degradation or inhibition of translation (Filipowicz et al., 2008; Meister, 2013; Ha and Kim, 2014). Canonical RNAi modulates gene expression by posttranscriptional gene silencing in the cytoplasm to regulate development, tumor suppression, and metabolism (He and Hannon, 2004; Calin and Croce, 2006). Human Dicer recognizes additional double-stranded (ds)RNA species, such as pre-mRNA, tRNA, and long noncoding RNA (Rybak-Wolf et al., 2014). Dicer also processes a subset of RNA polymerase II (RNAPII)-dependent, noncanonical miRNA precursors, which are termed *transcription start site miRNA* (Zamudio et al., 2014).

A growing body of evidence suggests that additional functions for Dicer proteins exist, which are independent of miRNA biogenesis and involve noncanonical modes of RNAi in the nucleus of various organisms (Castel and Martienssen, 2013). In fission yeast, nuclear Dcr1 facilitates transcriptional gene silencing of centromeric, heterochromatic repeats and repression of integrated transgenes by targeting dsRNA formed at actively transcribed loci (Provost et al., 2002; Volpe et al., 2002; Bühler et al., 2006). Dcr1 further promotes the release of RNAPII at termination regions of both highly transcribed protein-coding genes and antisense transcription units of tRNA and ribosomal RNA loci to resolve replication stress (Zaratiegui et al., 2011; Castel et al., 2014). Dicer has also various noncanonical functions in the nucleus of higher eukaryotes (Burger and Gullerova, 2015). Human nuclear Dicer modulates RNAPII transcription of coding and noncoding transcription units. Dicer stimulates RNAPII transcription at a subset of hormone-responsive promoters in complex with IFN-inducible, dsRNA-dependent protein kinase A activator and steroid-receptor RNA activator (Redfern et al., 2013), as well as silencing of the *secreted frizzled-related protein 1* (*SFRP1*) gene in cholangiocarcinoma cells (Cheng et al., 2017). We showed previously that human Dicer localizes to the nucleus to process endogenous (endo)-dsRNA derived from overlapping transcription units. In the absence of Dicer, unprocessed nuclear endo-dsRNA translocates to the cytoplasm and triggers IFN-mediated apoptosis (White et al., 2014). Formation of dsRNA around intronic polyadenylation sites recruits Dicer to chromatin to promote alternative polyadenylation (Neve et al., 2016). Dicer also generates endo-siRNA from dsRNA formed at terminator elements of protein-coding genes to guide heterochromatin formation. This leads to RNAPII pausing and promotes transcription termination (Skourti-Stathaki et al., 2014). Depletion of Dicer also impairs pre-mRNA processing (Haussecker and Proudfoot, 2005).

Recent findings link Dicer to the DNA damage response (DDR). Knockout of Dicer in the brain of developing mice causes accumulation of endogenous DNA damage and leads to cerebellar progenitor degeneration (Swahari et al., 2016a,b). Similarly, knockdown of Dicer in human HEK293 cells causes accumulation of DNA damage and triggers DNA damage signaling (Tang et al., 2008). Repair of DNA lesions by the DDR is crucial for genome stability (Jackson and Bartek, 2009; Cescutti et al., 2010). Although DNA double-strand breaks (DSBs) are repaired by homologous recombination and nonhomologous end joining (Wyman and Kanaar, 2006), additional mechanisms to target lesions involve changes in the chromatin landscape to increase accessibility of repair machineries (Hoeijmakers, 2001; Luijsterburg and van Attikum, 2011; Polo and Jackson, 2011). In response to UV irradiation, Dicer is recruited to DNA lesions to mediate chromatin decondensation during nt excision repair (Chitale and Richly, 2017). Moreover, DSBs trigger the accumulation of site-specific small noncoding RNA, termed DNA damage response RNA (DDRNA) in a Dicer-dependent manner in various organisms (Lee et al., 2009; Francia et al., 2012; Michalik et al., 2012; Wei et al., 2012). DDRNA facilitates recruitment of secondary repair factors, such as MDC1 and 53BP1, to establish DNA damage foci but are dispensable for recruitment of primary repair factors, such as the Mre11–Rad50–Nbs1 complex, which senses DNA lesions and initiates the DDR (Francia et al., 2016). DDRNA is also required for telomere maintenance (Rossiello et al., 2017).

A recent study challenged the existence of mouse Dicer in the nucleus itself (Much et al., 2016). Using a primary mouse fibroblast cell line, which expresses a catalytically active, endogenously tagged Dicer protein (HA::Dicer PMEF) at physiological levels (Comazzetto et al., 2014), Much et al. (2016) failed to detect any nuclear Dicer upon inhibition of nuclear export, DNA damage induction, or growth factor stimulation. These observations are in stark contrast to various other Dicer localization studies. We and others have shown that a subset human Dicer localizes to the nucleus in human cells (Passon et al., 2012; White et al., 2014) and is detected in nuclei devoid of cytoplasm (Khalil and Driscoll, 2010). Indeed, catalytically active Dicer has been purified from human nuclei (Gagnon et al., 2014). However, little is known about the regulatory principles that control nuclear Dicer function. Here, we show that multiple phosphorylation events regulate nuclear accumulation and activity of Dicer in response to DNA damage. Although phosphorylation of residue S1016 in the platform–Piwi/Argonaute/Zwille (PAZ)–connector helix is necessary and sufficient for Dicer nuclear accumulation, phosphorylation of carboxy-terminal residues S1728 and S1852 is required for efficient turnover of damage-induced dsRNA. Our data suggest a direct function of phosphorylated nuclear Dicer in the promotion of DNA repair in close proximity to DSBs.

## Results

### DNA damage-induced phosphorylation and accumulation of nuclear Dicer

The tumor suppressor p53 is an integral component of the DDR and has recently been shown to stimulate Dicer expression via the p53 family member TAp63. Although mutant TAp63 transactivates the *DICER1* locus, loss of p53 impairs Dicer expression (Su et al., 2010; Muller et al., 2014). This led us to test Dicer levels in human HEK293 cells subjected to DNA damage-inducing agents Etoposide (Eto; Hande, 1998), hydrogen peroxide, phleomycin, methyl methanesulfonate (MMS), or γ-irradiation. Surprisingly, Dicer expression was not significantly affected in HEK293 cells after continuous drug incubation (Fig. S1 A) or induction and repair of DNA damage (Fig. S1 B). Ser139 phosphorylation of the histone variant H2A.X (γH2A.X) was used as a marker for DNA damage.

**Figure 1.**
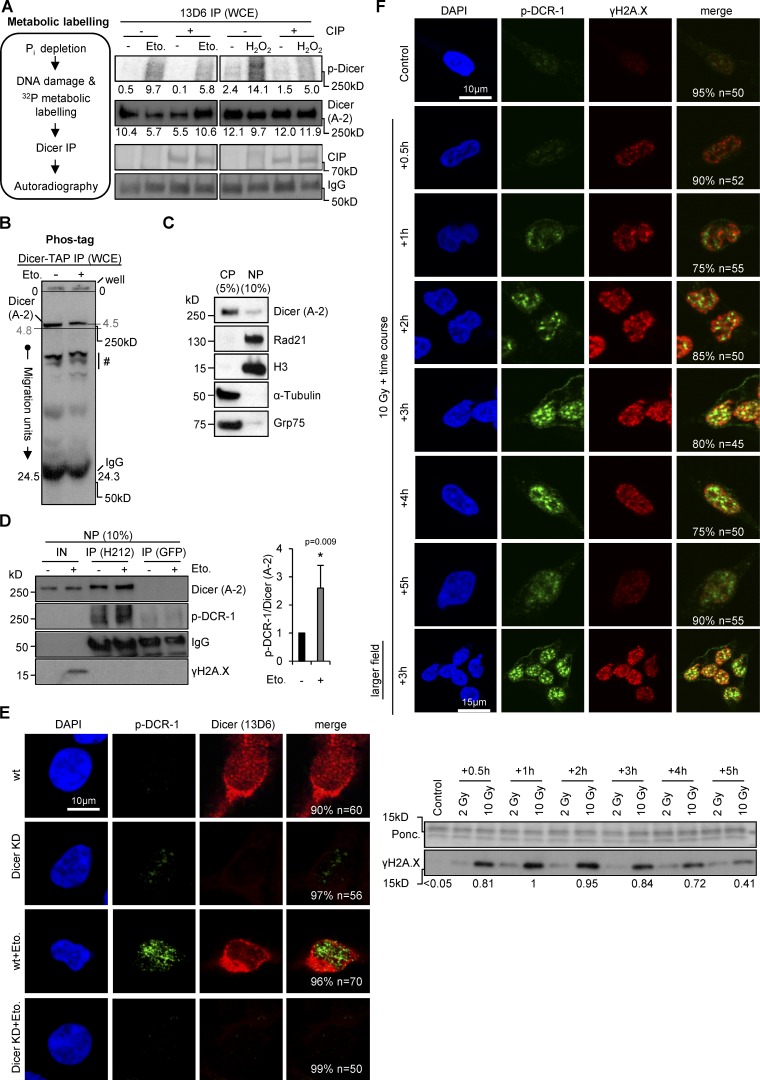
**Phosphorylation and nuclear accumulation of Dicer upon DNA damage in HEK293 cells.** (A) Detection of phosphorylated (autoradiograph, p-Dicer) or total Dicer (immunoblot, A-2) immunoprecipitated with 13D6 from whole cell extracts (WCE) after ^32^P-orthophosphate metabolic labeling in the absence or presence of calf intestine phosphatase (CIP). CIP signals, silver stain; Eto., etoposide; H_2_O_2,_ hydrogen peroxide; IgG, immunoglobulin heavy chain. Immunoblot signals were quantified using ImageJ. (B) Immunoblot showing Dicer-TAP migration by Phos-tag SDS-PAGE immunoprecipitated from whole cell extracts (WCE). IgG, immunoglobulin heavy chain; #, unspecific signal; migration units relative to wells. The entire gel is shown. (C) Immunoblots showing total Dicer (A-2) in subcellular fractions. CP/NP, cytoplasmic/nuclear fraction; fractionation marker: Rad21 and H3, nucleoplasm/chromatin (NP); α-tubulin, cytoplasm (CP); Grp75, mitochondria. (D) Immunoblots detecting phosphorylated histone variant H2A.X (γH2A.X, S139), total (A-2) and phosphorylated (p-DCR-1) endogenous Dicer immunoprecipitated from nuclear lysates using the H212 antibody. GFP, control immunoprecipitation (IP; left). Quantitation of p-DCR-1 IP signals as fold-change over total Dicer IP signals (right). *, P < 0.05; error bars, means ± SEM of three biological replicates. (E) Confocal imaging of phosphorylated (p-DCR-1) and total (13D6) Dicer in wild-type or Dicer-depleted (Dicer KD) cells. All quantifications represent number of cells that have the shown phenotype. (F) Confocal imaging as in E (top) and immunoblots (bottom) of phosphorylated (p-DCR-1) Dicer and γH2A.X after time course kinetics with γ-irradiation. Ponc., Ponceau S staining, loading control; Gy, Gray.

We speculated that DNA damage might alter posttranslational modifications of Dicer. To assess changes in Dicer phosphorylation in response to DNA damage, we performed [^32^P]orthophosphate in vivo metabolic labeling before immunoprecipitation of endogenous Dicer in wild-type HEK293 cells (Fig. 1 A). We detected 5–10-fold induction of various damage-inducible and phosphatase-sensitive bands migrating at ∼250 kD. We further observed a shift in migration of Dicer, but not immunoglobulin heavy chain by 6.2% on Phos-tag gels after immunoprecipitation of tandem affinity purification (TAP)–tagged Dicer from cells treated with Eto (Fig. 1 B).

To assess the subcellular distribution of Dicer upon DNA damage, we used subcellular fractionation of HEK293 cells (Fig. 1 C) and a previously characterized phospho-specific Dicer antibody (p-DCR-1), which recognizes two conserved phospho-serine residues (S1728 and S1852; Drake et al., 2014). We detected a two- to threefold increase in p-DCR-1, but not total Dicer signal in damaged nuclei after immunoprecipitation of endogenous Dicer (Fig. 1 D) or TAP-tagged Dicer (Fig. S1 C). We confirmed specific enrichment of TAP-tagged Dicer in cells lacking endo-Dicer by comparison with background in noninduced cells (Fig. S1 D). Using confocal microscopy, we detected several p-DCR-1 spots after incubation with Eto (Fig. S1 E). To monitor the specificity of the p-DCR-1 antibody, we made use of a conditional Dicer-knockdown cell line (Schmitter et al., 2006). Depletion of Dicer was confirmed by staining with the 13D6 antibody, which recognizes total Dicer. Moreover, p-DCR-1 foci were only visible in the nuclei of damaged, wild-type, but not Dicer-depleted, HEK293 cells upon incubation with Eto (Fig. 1 E) or hydrogen peroxide (Fig. S1 F). Next, we applied γ-irradiation and detected a wave of nuclear p-DCR-1 staining concomitant with induction and clearance of γH2A.X using time kinetics. Phosphorylation of H2A.X was strongly induced after 30 min and remained high up to 3 h after irradiation (Fig. 1 F). In contrast, p-DCR-1 did not stain cells treated with osmotic stress (0.1× PBS, 10× PBS) or hydroxyurea (HU; Fig. S1 G). Hydroxyurea induces γH2A.X originating from stalled replication forks (Ward and Chen, 2001) and stimulates phosphorylation of ataxia telangiectasia mutated (ATM)/ATM-related (ATR) kinase substrates (Fig. S1 H), suggesting that nuclear p-DCR-1 foci are primarily caused by DSBs. We further measured proliferation of damaged HEK293 cells and monitored expression of cellular markers of proliferation (Ki-67) and apoptosis (cleaved poly-ADP-ribose polymerase, PARPc) to rule out that nuclear Dicer activity is primarily caused by induction of apoptosis, as demonstrated previously in *Caenorhabditis elegans* (Nakagawa et al., 2010). Unlike staurosporine (STS), an apoptosis-inducing kinase inhibitor (Kabir et al., 2002), incubation with DNA-damaging agents for 2 h did not significantly alter proliferation (Fig. S1 I) or expression of Ki-67 or levels of PARPc, but induced γH2A.X (Fig. S1 J). Surprisingly, we could not detect significant damage-induced changes in the subcellular localization of Dicer with antibodies that recognize the total Dicer pool in immunoblotting (A-2) and confocal imaging (13D6) experiments. We conclude that a fraction of the cellular Dicer pool is responsive to DNA damage and accumulates in the nucleus upon phosphorylation.

### Recruitment of phosphorylated Dicer (p-Dicer) to DNA DSBs

**Figure 2.**
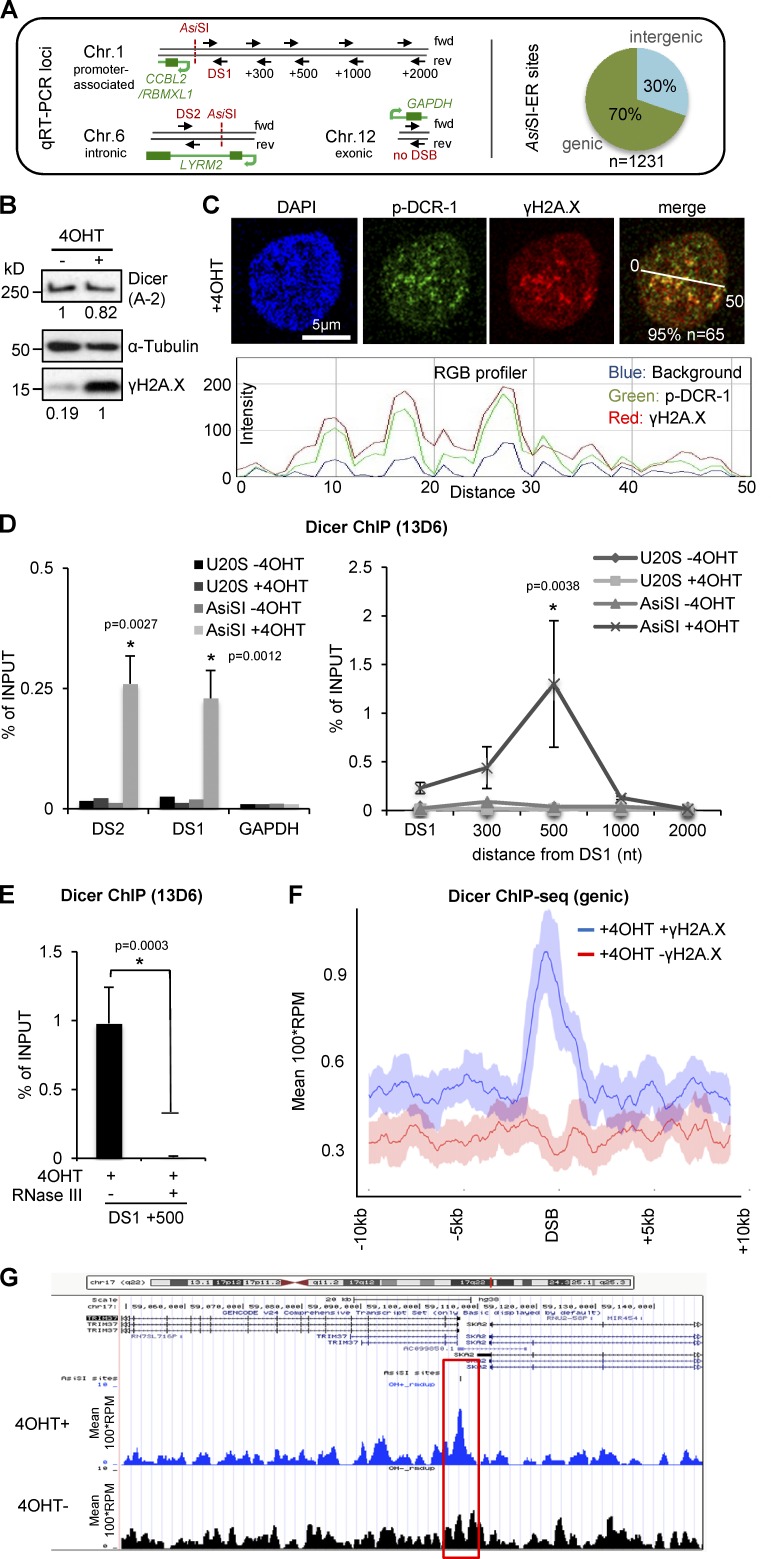
**Recruitment of Dicer to DNA double-strand breaks in *Asi*SI-ER U2OS cells.** (A) Structure of genomic loci assessed by quantitative RT-PCT (left) and genome-wide *Asi*SI-ER target site distribution; n, number of predicted *Asi*SI-ER target sites (right); fwd/rev, forward/reverse. (B) Immunoblots detecting total Dicer (A-2) and γH2A.X after induction of DNA double-strand breaks (DSBs). 4OHT, 4-hydroxytamoxifen. Immunoblots were quantified using ImageJ. (C) Confocal imaging of phosphorylated Dicer (p-DCR-1) and γH2A.X (top). All quantifications represent number of cells exhibiting shown phenotype. Quantification using ImageJ RGB profiler (bottom). (D) ChIP analysis showing Dicer occupancy at DSBs DS1/2 in wild-type and *Asi*SI-ER U2OS cells using site-specific primers. GAPDH, control locus. *, P < 0.05; error bars, means ± SEM of three biological replicates. (E) ChIP analysis showing Dicer occupancy at DS1 in absence or presence of recombinant RNase III preincubation. *, P < 0.05; error bars, means ± SEM of three biological replicates. (F) ChIP-seq signal upon +4OHT incubation at 200 γH2A.X-positive/negative genic sites after removal of duplicate reads. A rolling mean of 1 kb was applied after removal of 2% of the top and bottom values. Shadow, rolling SD. (G) Snapshot showing Dicer binding at genic *Asi*SI target site upstream of *TRIM37* before (4OHT^−^) and after (4OHT^+^) DNA damage. Red box, proximal region to *Asi*SI site.

Recent findings indicate that Dicer promotes DNA repair by generating site-specific, small regulatory RNA in close proximity to DSBs in various organisms (Francia et al., 2012; Michalik et al., 2012; Wei et al., 2012). To assess involvement of human Dicer at DSBs, we used the *Asi*SI-ER U2OS cell line, which harbors the recombinant endonuclease, *Asi*SI, which is fused to the estrogen receptor (ER) ligand-binding domain (Iacovoni et al., 2010). Treatment with 4-hydroxytamoxifen (4OHT) triggers nuclear translocation of *Asi*SI-ER and induces DSBs at *Asi*SI target sites (GCGAT|CGC, nonmethylated), which allows sequence-specific analysis of DSB-associated proteins. First, we confirmed inducible γH2A.X chromatin immunoprecipitation (ChIP) signals at two previously characterized *Asi*SI sites (DS1, chr1:88993018–88993227; *CCBL2/RBMXL1* promoter; DS2, chr6:89638287–89638451, *LYRM2* intron 1; Caron et al., 2012; Fig. 2 A, left). The human genome contains 1,231 predicted *Asi*SI-ER targets sites in both genic and intergenic regions (Fig. 2 A, right). We detected strong, 4OHT-inducible γH2A.X ChIP signals at DS1/2 and up to 1-kb distant from DS1 in *Asi*SI-ER U2OS cells, but not in wild-type U2OS cells or at a nontargeted, exonic *GAPDH* control locus (Fig. S2 A). In line with DNA-damaging agents, we confirmed a 4OHT-dependent induction of γH2A.X, but not Dicer levels, by immunoblotting (Fig. 2 B) and partial colocalization of Dicer with γH2A.X-positive damage foci (Fig. 2 C).

Our p-DCR-1 data suggest that Dicer localizes in close proximity to γH2A.X in damaged nuclei. To test recruitment of Dicer to DSBs, we used ChIP analysis at DS1/2 using the 13D6 antibody. Strikingly, we detected a four- to sixfold increase in Dicer occupancy upon induction of DSBs at DS1/2 (Fig. 2 D, left). Dicer recruitment peaked ∼500-nt distant from DS1 (Fig. 2 D, right) and was sensitive to preincubation with recombinant, dsRNA-specific RNase III (Fig. 2 E) as well as depletion of endogenous Dicer by transiently transfected shRNA (Fig. S2, B and C). To assess Dicer chromatin occupancy at DSBs globally, we used Dicer ChIP-seq analysis in *Asi*SI-ER U2OS cells. Meta-gene analysis revealed genome-wide Dicer association with γH2A.X-positive, *Asi*SI-restricted DSBs at genic loci, such as the *TRIM37* promoter (Fig. 2, F and G; and Fig. S2 D) upon 4OHT incubation. Dicer levels were not increased at various *Asi*SI sites in control HEK293 cells (Fig. S2 E). Dicer occupancy was increased two- to threefold at restricted genic *Asi*SI target sites, but was also detectable at intergenic loci (Fig. S2, F and G) upon DNA damage induction. During cell division and nuclear membrane disassembly, a fraction of the *Asi*SI enzyme can leak into the nucleus in absence of 4OHT to target highly accessible *Asi*SI sites. This phenomenon can cause a certain “damage-like phenotype” in −4OH conditions, especially in genome wide analyses. We conclude that Dicer is recruited to DSBs in a dsRNA-dependent manner.

Next, we assessed whether DNA damage signaling induces Dicer phosphorylation at residues S1728 and S1852. Three members of the phosphatidylinositol-3-kinase (PI3K) family, ATM, ATR, and DNA-dependent protein kinase (DNA-PK) govern the response to DNA damage by phosphorylating hundreds of substrates (Kastan and Lim, 2000; Matsuoka et al., 2007; Giglia-Mari et al., 2011; Maréchal and Zou, 2013). We speculated that PI3Ks target Dicer in the DDR. Indeed, preincubation of *Asi*SI-ER U2OS cells with various kinase inhibitors prevented damage-induced p-DCR-1 foci formation and accumulation of γH2A.X but did not affect Dicer expression (Fig. S3, A and B). Similarly, p-DCR-1 foci were largely diminished after depletion of the DNA-PK catalytic subunit (Fig. S3, C and D). We conclude that DSB-induced Dicer phosphorylation at residues S1728 and S1852 is dependent on PI3K signaling.

### Phosphorylation of Dicer residue S1016 is necessary and sufficient for nuclear localization

**Figure 3.**
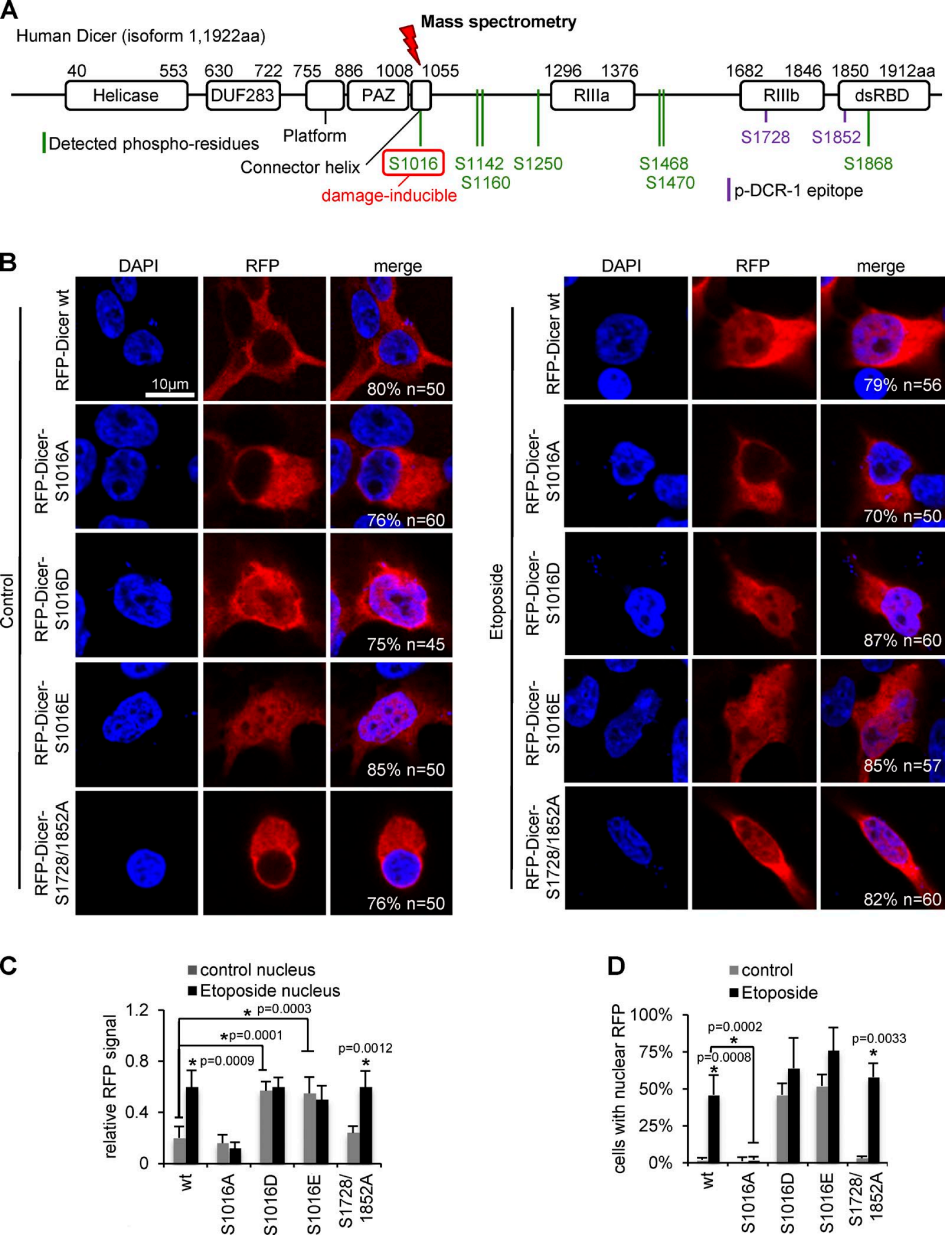
**Nuclear accumulation of S1016 phosphorylated Dicer upon DNA damage in HEK293 cells.** (A) Schematic of human Dicer isoform 1 (NP_001258211.1) domain structure and positions of assessed phosphorylated residues. DUF283, domain of unknown function; PAZ, Piwi/Argonaute/Zwille; RIIIa/b, RNase III a/b; dsRBD, double-stranded RNA binding domain. (B) Confocal imaging of RFP-tagged Dicer constructs expressed in wild-type HEK293 cells. All quantifications represent the number of cells that have the shown phenotype. (C) Relative quantification of (B). Bars, mean ratio (nuclear/cytoplasmic RFP) normalized to the background, *n* > 45. (D) Absolute quantification of (B). Bars, mean number of cells with nuclear RFP signal, *n* > 45. *, P < 0.05.

More than 30 phosphoresidues have been detected for human Dicer (http://www.phosphosite.org). To analyze DNA damage-induced Dicer phosphorylation in detail, we used comparative phosphoproteomics of total Dicer immunoprecipitated from HEK293 nuclei. In total, we detected seven phosphorylated Dicer residues. A single serine residue in the Dicer platform–PAZ–connector helix S1016 was increased threefold upon DNA damage, whereas unmodified Dicer peptides did not change (Fig. 3 A, Fig. S4, and Tables S1 and S2). These findings suggest that a subset of Dicer is phosphorylated upon DNA damage at residue S1016. Surprisingly, we could not detect phosphorylation of serine residues 1728 or 1852 phosphopeptides, which correspond to the p-DCR-1 epitope.

To assess the relevance of Dicer phosphorylation for subcellular localization, we created RFP-tagged nonphosphorylatable (residues S1016A, S1728/1852A) or phosphomimetic (S1016D, S1016E) Dicer mutants and expressed them in wild-type HEK293 cells (Fig. 3 B and Fig. S5 A). Although RFP-Dicer^wt^ and RFP-Dicer^S1016A^ localized mostly in the cytoplasm in nondamaged cells, RFP-Dicer^wt^, but not RFP-Dicer^S1016A^, displayed increased nuclear accumulation upon Eto treatment in a subset of cells. In contrast, RFP-Dicer^S1016D/E^ displayed consistent nuclear localization. Surprisingly, the RFP-Dicer^S1728/1852A^ double mutant remained nuclear (Fig. 3, C and D). We confirmed comparable expression of all RFP constructs in these cells (Fig. S5 B). Similarly, we detected damage-induced nuclear localization of GFP-tagged, wild-type Dicer in a subset of cells (Fig. S5 C).

So far, we assessed RFP-Dicer localization after overexpression of tagged-Dicer and in presence of endo-Dicer. Although detecting clear differences in subcellular localization, we next aimed to express the RFP-Dicer constructs in Dicer-depleted (Dicer KD) cells to assess damage-induced Dicer localization in the absence of endo-Dicer (Fig. S5 D). Note that the shRNA, which targets endo-Dicer, prevents overexpression of RFP-Dicer constructs in Dicer KD cells, resulting in more physiologic expression levels (compare Fig. S5, B and E). Consistently, we detected nuclear RFP-Dicer^wt^ upon damage. When expressing RFP-Dicer^S1016A^ in damaged Dicer KD cells, the p-DCR-1 antibody displayed strong cytoplasmic signals, colocalizing with nonphosphorylatable RFP-Dicer^S1016A^. In contrast, expression of the RFP-Dicer^S1728/1852A^ double mutant displayed nuclear RFP signal but no detectable p-DCR-1 signal, underscoring the specificity of the p-DCR-1 antibody. We conclude that phosphorylation of residue S1016, but not S1728/S1852, is necessary and sufficient for nuclear accumulation of Dicer.

### Damage-induced accumulation of nuclear Dicer is conserved in mammals

**Figure 4.**
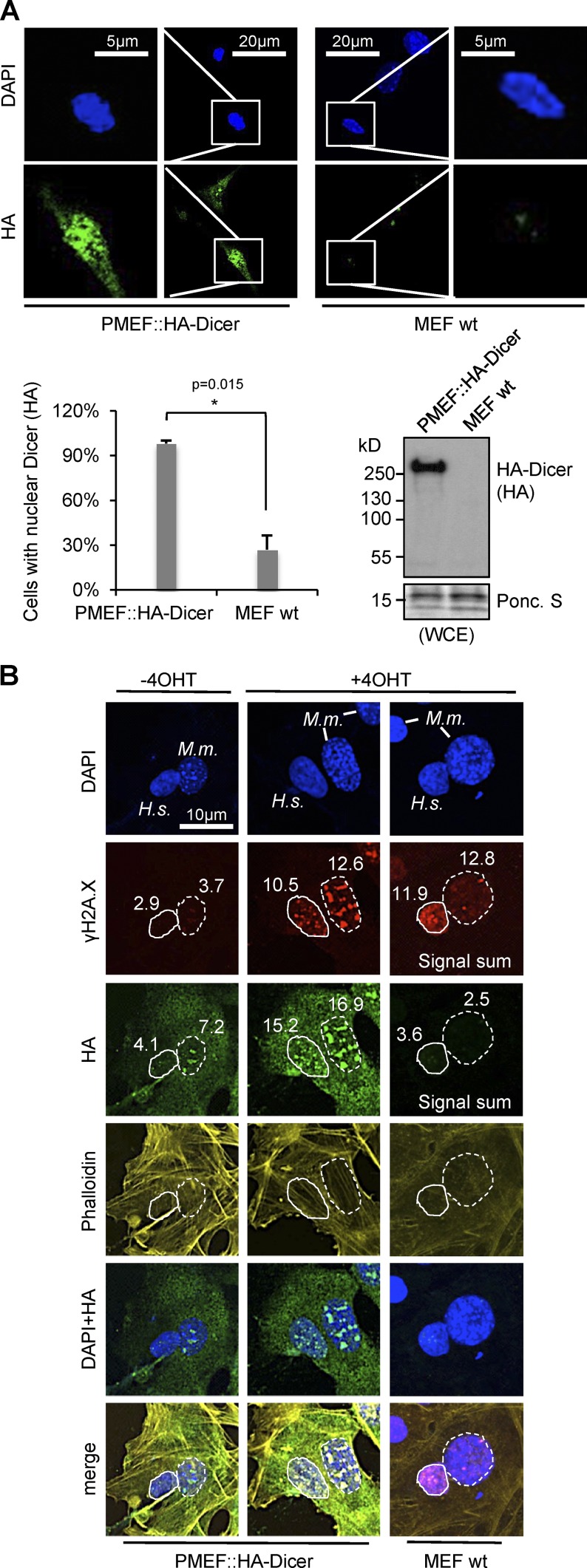
**Accumulation of mouse HA-Dicer in damaged nuclei.** (A) Confocal imaging (top) detecting endogenously tagged, mouse HA-Dicer in primary MEF cell line PMEF::HA-Dicer. Control, MEF wild type. Quantitation as the percentage of cells with nuclear HA signal. *n* > 50 (bottom left). *, P < 0.05; error bars, means ± SEM of three biological replicates and immunoblot detecting HA-Dicer (bottom right). WCE, whole cell extract; Ponceau S staining, loading control. (B) Confocal imaging of heterokaryon fusions between human *Asi*SI-ER U2OS cells and mouse PMEF::HA-Dicer or wild-type MEF cells, respectively. Quantitations are shown as signal sums (mean intensity × area). Nuclei: H.s., *Homo sapiens*, full circle; M.m., *Mus musculus*, dashed circle. Number of analyzed fused cells *n* > 15.

Encouraged by DNA damage-induced nuclear Dicer accumulation in HEK293 cells, we next investigated the subcellular localization of endogenously tagged Dicer. Therefore, we used a recently described HA-tagged mouse embryonic fibroblast (MEF) cell line (PMEF::HA-Dicer; Comazzetto et al., 2014). First, we assessed the specificity of the HA antibody and confirmed expression of full length HA-tagged Dicer in PMEF::HA-Dicer cells by confocal imaging and immunoblotting (Fig. 4 A). Surprisingly, HA staining was detectable in >90% of PMEF::HA-Dicer nuclei, in addition to widespread cytoplasmic staining. The HA reactivity was largely diminished in wild-type MEF cells and generated a single band migrating at ∼250 kD after incubation with PMEF::HA-Dicer, but not wild-type, MEF extracts.

Next, we performed an interspecies heterokaryon assay to assess changes in the subcellular localization of mouse HA-Dicer in response to *Asi*SI-ER–induced DSBs. Co-culture and fusion of human *Asi*SI-ER U2OS cells with either wild-type or HA-Dicer–expressing MEFs resulted in sporadic formation of interspecies heterokaryons, consisting of a cytoplasmic continuum with both human and mouse nuclei (Fig. 4 B). In absence of 4OHT, we could detect neither significant induction of γH2A.X nor nuclear accumulation of HA-Dicer in human nuclei. In mouse nuclei, γH2A.X levels were also low and accompanied by modest HA staining. Strikingly, addition of 4OHT strongly elevated γH2A.X signals, confirming DSB induction by the *Asi*SI-ER endonuclease in both human and mouse nuclei. Concomitantly, we detected strong, spotted HA staining in nuclei of both species. HA signals colocalized with γH2A.X-positive foci, suggesting recruitment of mouse HA-Dicer to human DSBs. We noticed that the *Asi*SI-ER endonuclease encoded in U2OS cells was also HA tagged. To dissect the contribution of HA-Dicer and *Asi*SI-ER toward the observed HA staining, we fused wild-type MEF cells with *Asi*SI-ER U2OS cells, resulting in interspecies heterokaryons devoid of HA-Dicer. We observed no HA signal in mouse nuclei, despite induction of γH2A.X foci in the presence of 4OHT. Similarly, colocalization of γH2A.X foci with HA signals was also greatly reduced in human nuclei. We conclude that the HA staining observed in interspecies heterokaryons mostly represents mouse HA-Dicer and that damage-induced nuclear Dicer localization and recruitment to DSBs is conserved in mammals.

### Processing of damage-induced dsRNA by nuclear Dicer

**Figure 5.**
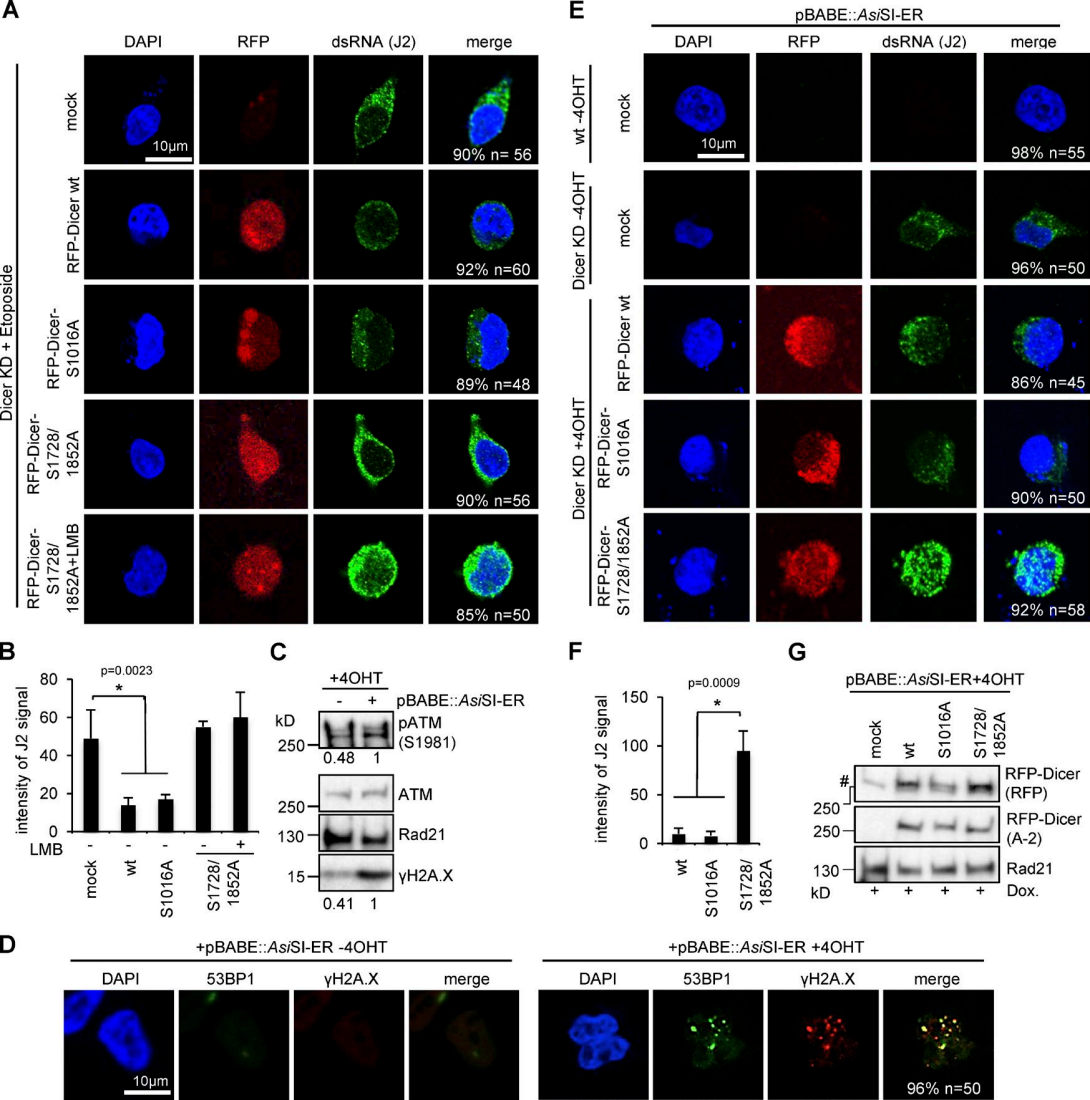
**Prerequisite of Dicer S1728/1852 phosphorylation for damage-induced dsRNA processing in HEK293 cells.** (A) Confocal imaging of RFP-tagged Dicer constructs and dsRNA (J2) in Dicer-depleted (Dicer KD) cells transfected with RFP-Dicer constructs in the absence or presence of Leptomycin B (LMB). *n* > 30. Control, mock transfected Dicer KD cells. See also Fig. S5 F for additional controls. All quantifications represent the number of cells that have the shown phenotype. (B) Quantification of dsRNA from A using ImageJ. J2 signal was normalized to the background. *, P < 0.05; error bars, means ± SEM of three biological replicates. (C) Immunoblots detecting ataxia telangiectasia mutated (ATM) or phospho-ATM (S1981) and γH2A.X in the absence or presence of *Asi*SI-ER expression vector pBABE::*Asi*SI-ER in wild-type HEK293 cells. (D) Confocal imaging of 53BP1 and γH2A.X in wild-type cells transfected with recombinant *Asi*SI-ER expression vector pBABE::*Asi*SI-ER. All quantifications represent the number of cells that have the shown phenotype. (E) Confocal imaging of RFP-tagged Dicer constructs and dsRNA (J2) in Dicer-depleted (Dicer KD) cells cotransfected with RFP-Dicer constructs and pBABE::*Asi*SI-ER. Controls, mock transfected wild-type or Dicer KD cells. All quantifications represent the number of cells that have the shown phenotype. (F) Quantification of dsRNA, *, P < 0.05; error bars, means ± SEM of three biological replicates; from E using ImageJ. J2 signal normalized to background. (G) Immunoblots detecting expression of RFP-Dicer constructs (RFP) in wild-type and Dicer-depleted HEK293 cells cotransfected with pBABE::*Asi*SI-ER. Dox., doxycycline; Rad21, loading control; #, unspecific signal.

We noticed that Dicer ChIP signals were sensitive to RNase III incubation in vitro and speculated that Dicer might recognize damage-induced dsRNA as a substrate in vivo. To assess the effect of Dicer phosphorylation on dsRNA processing, we transfected Dicer KD cells with RFP-Dicer^wt^, RFP-Dicer^S1016A^, or RFP-Dicer^S1728/1852A^ and visualized dsRNA levels using the dsRNA-specific antibody J2. We and others previously have confirmed the specificity of J2 toward long dsRNA (>40 bp), but not hairpin pre-miRNA or single-stranded RNA in vitro and in vivo (Bonin et al., 2000; Weber et al., 2006; White et al., 2014). Although no significant onset of J2 reactivity was detectable in damaged wild-type HEK293 cells (Fig. S5 F), incubation with Eto caused cytoplasmic accumulation of dsRNA in untransfected, Dicer KD cells (Fig. 5 A). Processing of dsRNA was partially restored by expression of RFP-Dicer^wt^ and RFP-Dicer^S1016A^, resulting in a two- to threefold decrease in J2 signal intensity (Fig. 5 B). In contrast, RFP-Dicer^S1728/1852A^ failed to process dsRNA and dsRNA levels accumulated in the cytoplasm, resembling mock-transfected cells. A modest accumulation of nuclear J2 signal was detected in Dicer KD cells transfected with RFP-Dicer^S1728/1852A^ after preincubation with the nuclear export inhibitor leptomycin B (LMB), suggesting that damage-induced dsRNA originates in the nucleus. To confirm that Dicer is specifically required for dsRNA processing at DSBs, we induced sequence-specific DSBs in HEK293 cells by transient transfection of recombinant *Asi*SI-ER endonuclease. First, we monitored induction of DSBs in HEK293 cells. 4OHT incubation caused a twofold induction of both S1981-phosphorylated ATM kinase and γH2A.X, two hallmarks of DNA damage (Fig. 5 C). To exclude induction of γH2A.X being due to cellular stress caused by plasmid transfections, we tested for 53BP1-positive damage foci in HEK293 cells transfected with pBABE::*Asi*SI-ER plasmid (Fig. 5 D). Indeed, 4OHT induced several foci containing both 53BP1 and γH2A.X, strongly suggesting that *Asi*SI-ER generates DSBs in HEK293 cells. For proof of principle, we transfected Dicer KD cells with RFP-Dicer^wt^, RFP-Dicer^S1016A^, or RFP-Dicer^S1728/1852A^ and assessed dsRNA levels (Fig. 5 E). Again, we demonstrate impaired nuclear accumulation of nonphosphorylatable RFP-Dicer^S1016A^ and impaired processing of damage-induced dsRNA by reconstitution with nonphosphorylatable RFP-Dicer^S1728/1852A^, as visualized by a 5–10-fold accumulation of dsRNA in both the cytoplasm and the nucleus (Fig. 5 F). Importantly, J2 reactivity was not detected in nondamaged, mock-transfected, wild-type HEK293 cells but was increased modestly upon Dicer depletion. We also confirmed comparable expression levels of RFP-Dicer constructs (Fig. 5 G). We noticed an apparent discrepancy in the pattern of damage-induced dsRNA accumulation. After expression of the nonphosphorylatable Dicer S1728/1852A double mutant in Dicer KD cells, we found Eto-induced dsRNA accumulating primarily in the cytoplasm, whereas *Asi*SI-ER cleavage increased both nuclear and cytoplasmic J2 reactivity (compare J2 signal in Fig. 5, A and E). We suspect this is due to a different quality of DNA-damage induction. The topoisomerase II inhibitor etoposide causes a rapid, global, and saturated induction of DSBs, generating high levels of γH2A.X after 2 h, whereas *Asi*SI-ER-induced damage generates only a fraction of the amount of DSBs, targeting several hundred loci within 4 h (compare γH2A.X in Fig. S1 E and Fig. 2 C). We conclude that nuclear Dicer processes damage-induced dsRNA if it is catalytically active, which was the case for RFP-Dicer, wild-type cells. RFP-Dicer S1016A was also catalytically active but could not relocate to the nucleus. Thus, in the case of S1016A, aberrant, nonprocessed, damage-induced nuclear dsRNA is exported to the cytoplasm, where RFP-Dicer S1016A processes it. In contrast, RFP-Dicer S1728/S1852 can localize to the nucleus but is catalytically impaired. Aberrant dsRNA escapes nuclear processing and accumulates in the cytoplasm. We further conclude that DSB-induced phosphorylation of Dicer residue S1016 is necessary and sufficient for nuclear accumulation, whereas phosphorylation of S1728/1852 residues is required for dsRNA processing in the nucleus.

### Accumulation of DNA damage in Dicer-depleted cells

**Figure 6.**
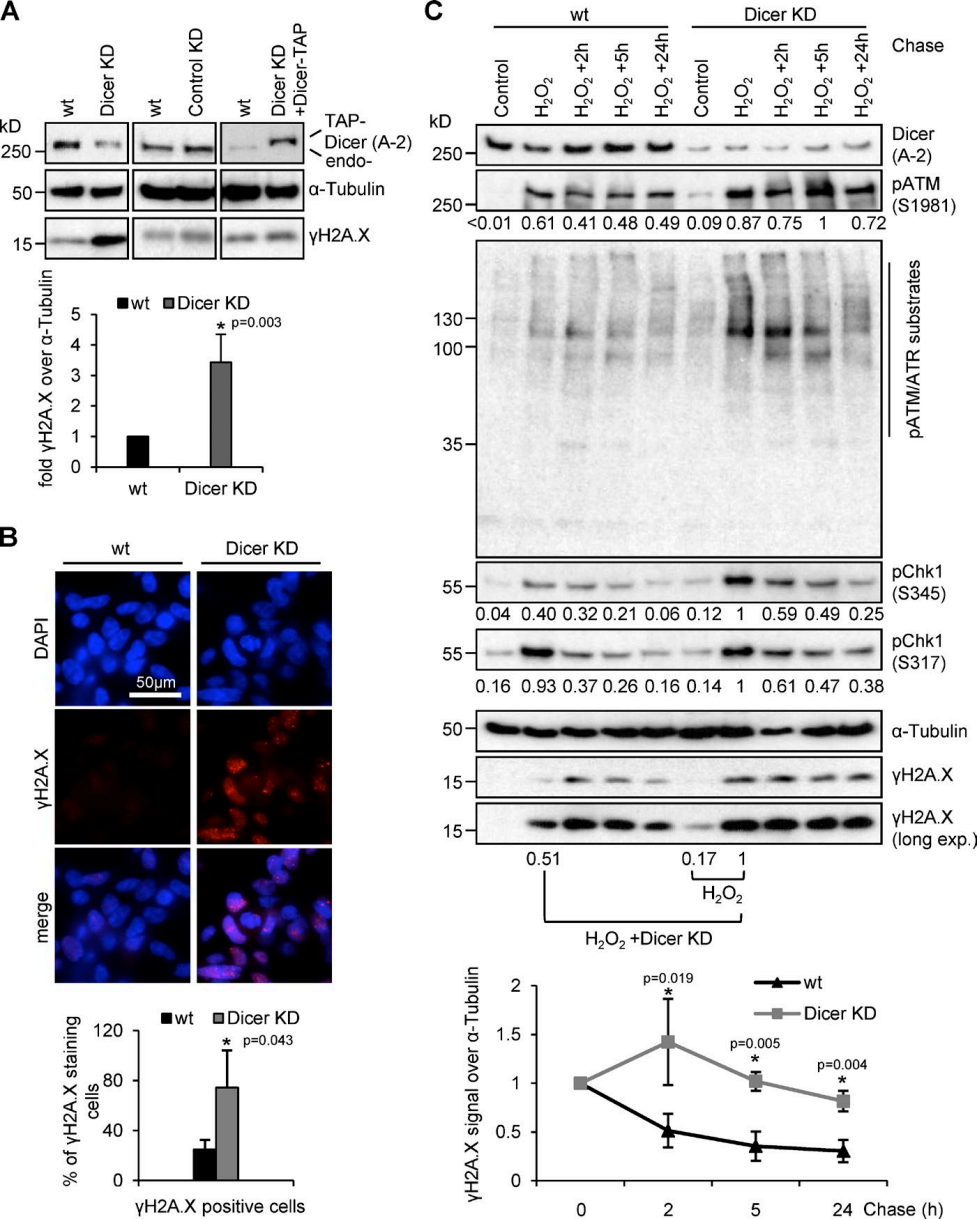
**Endogenous DNA damage and delayed repair in Dicer-depleted HEK293 cells.** (A) Immunoblots detecting total Dicer (A-2) and γH2A.X levels after Dicer depletion (Dicer KD), expression of a scrambled shRNA (control KD) or reexpression of TAP-tagged Dicer (top). Quantitation of γH2A.X levels in wild-type and Dicer KD cells (bottom), *, P < 0.05; error bars, means ± SEM of three biological replicates. (B) Epifluorescence imaging of γH2A.X staining in wild-type and Dicer KD cells (top). Quantitation of γH2A.X levels as the percentage of γH2A.X-positive cells, *n* > 200 (bottom); *, P < 0.05; error bars, means ± SEM of three biological replicates. (C) Immunoblots detecting total Dicer (A-2), phospho-ATM (S1981), substrates of ATM/ATR phosphorylation, phospho-Chk1 (S345, S317), and γH2A.X after pulse-chase treatment with hydrogen peroxide (H_2_O_2_) in wild-type and Dicer KD cells (top). Quantitation of γH2A.X levels using ImageJ (bottom), *, P < 0.05; error bars, means ± SEM of three biological replicates.

Next, we wished to test the relevance of Dicer for DNA repair. Using the conditional Dicer knockdown system, we detected three- to fourfold elevated levels of γH2A.X in Dicer KD cells, which were rescued by Dicer-TAP reconstitution (Fig. 6 A). We further observed a two- to threefold increase in γH2A.X-positive foci (Fig. 6 B). Importantly, conditional Dicer depletion has no significant effect on steady-state mRNA levels for most DNA repair factors (Schmitter et al., 2006). γH2A.X is a hallmark of replication stress, and nuclear Dicer has been linked to removal of stalled replication forks in *Schizosaccharomyces pombe* (Castel and Martienssen, 2013). To rule out that elevated γH2A.X levels in the absence of Dicer represent primarily stalled replication forks, we assessed the cell cycle distribution of Dicer KD cells (Fig. S5 G). Quantitation of the cell-cycle distribution revealed no significant change after Dicer depletion. Instead, we observed that Dicer depletion caused prolonged phosphorylation of DNA damage-responsive kinases ATM and Chk1, as well as delayed clearance of phosphorylated ATM/ATR substrates and γH2A.X levels (Fig. 6 C). Moreover, the combination of Dicer depletion with hydrogen peroxide caused an additive increase in γH2A.X levels. We conclude that accumulation of γH2A.X levels upon Dicer depletion primarily represents induction of DNA damage and that the DDR is delayed in the absence of Dicer.

**Figure 7.**
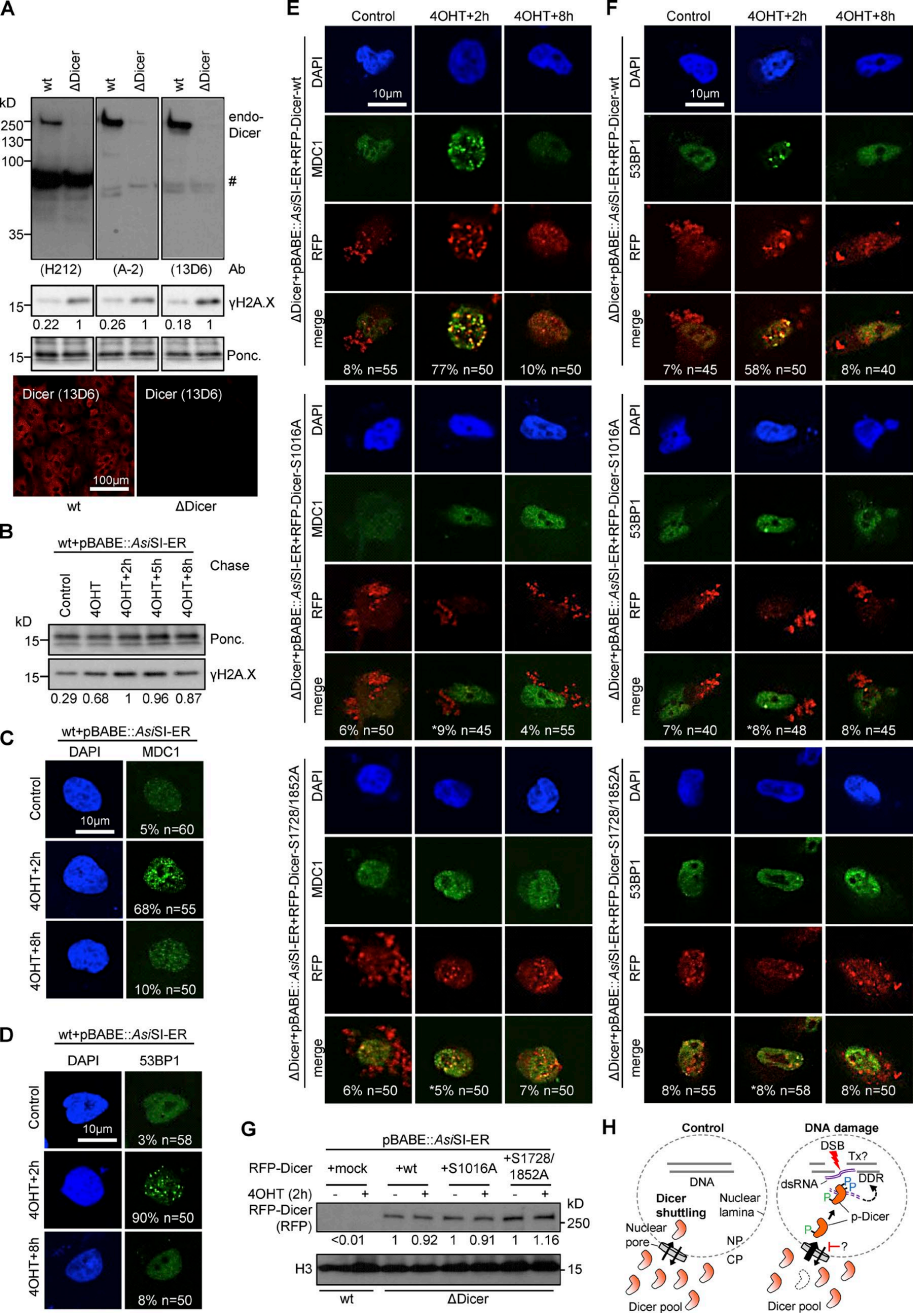
**Impaired recruitment of DNA repair factors upon mutation of Dicer in A549 cells.** (A) Immunoblots (top) and confocal microscopy (bottom) detecting endogenous Dicer (H212, A-2, 13D6) in wild-type and Dicer knockout (ΔDicer) A549 cells. Ponc., Ponceau S, loading control; #, unspecific signal. (B) Immunoblots detecting γH2A.X levels in wild-type A549 cells after transfection with pBABE::*Asi*SI-ER plasmid and 4OHT incubation (2 h pulse). (C and D) Confocal imaging of MDC1 (C) and 53BP1 (D) in wild-type A549 cells after transfection with pBABE::*Asi*SI-ER plasmid and 4OHT incubation as indicated. (E and F) Imaging as in C and D, but performed in ΔDicer cells, including transfection of RFP-Dicer constructs. All quantifications represent the percentage of foci positive cells, *n* = number of cells analyzed. *, P < 0.05; error bars, means ± SEM of three biological replicates. (G) Immunoblots detecting expression of RFP-Dicer constructs in the absence or presence of 4OHT. (H) Model for DNA damage-induced redistribution of the cellular Dicer pool. In undamaged cells (control), Dicer is a predominantly a cytoplasmic protein that shuttles to the nucleus sporadically and is rapidly exported back to the cytoplasm (CP). In the presence of DSBs, the DNA damage response (DDR) targets a small fraction of the cellular Dicer pool by arguably sequential phosphorylation of serine residues S1016 (green) and S1728/S1852 (blue), which causes accumulation in the nucleoplasm (NP) and recruitment to DSBs. Phosphorylated Dicer (p-Dicer) binds and processes dsRNA, which may be produced by RNAPII transcription at lesions to promote the DDR. Phosphorylation of Dicer at S1016 may also alter the import/export rate.

To test the relevance of Dicer residues S1016, S1728, and S1852 for the DDR in absence of endo-Dicer, we generated a human A549 Dicer knockout cell line (▵Dicer) using CRISPR/Cas9 and validated both the loss of Dicer expression and the accumulation of γH2A.X in ΔDicer cells (Fig. 7 A). Next, we transfected wild-type A549 cells with pBABE::*Asi*SI-ER to test for induction of DSBs. Indeed, we observed a wave of γH2A.X induction, peaking 2 h after removal of 4OHT (Fig. 7 B), and time-dependent formation of damage foci, positive for DSB repair factors MDC1 (Fig. 7 C, percentage of MDC1 foci–positive cells) and 53BP1 (Fig. 7 D, percentage of 53BP1 foci–positive cells). Next, we co-transfected pBABE::*Asi*SI-ER and RFP-Dicer constructs into ▵Dicer cells. Similar to wild-type A549 cells, we observed formation of MDC1- and 53BP1-positive foci after 4OHT incubation and complementation with RFP-Dicer^wt^ (Fig. 7, E and F, showing percentage of foci-positive cells; for quantification of foci intensity signal, see Fig. S5 H). Reassuringly, nuclear RFP-Dicer^wt^ partially colocalized with damage foci. After transfection of RFP-Dicer^S1016A^ or RFP-Dicer^S1728/1852A^, however, recruitment of both MDC1 and 53BP1 to the damage foci was largely impaired. We also observed morphological changes, arguably caused by increased cellular stress, in ΔDicer cells complemented with nonphosphorylatable RFP-Dicer mutants. Finally, we confirmed comparable expression of RFP-Dicer constructs in ΔDicer cells (Fig. 7 G). We conclude that wild-type, nuclear Dicer, phosphorylated both at residues S1016 and S1728/S1852, promotes recruitment of DNA repair factors MDC1 and 53BP1 to DSBs.

Collectively, we unravel a damage-inducible Dicer phosphoswitch to engage a subset of cellular Dicer in nuclear dsRNA processing in close proximity to DSBs to promote the DDR (Fig. 7 H).

## Discussion

Our data provide novel insights into Dicer function during the DDR. We identify a damage-inducible phosphoswitch at human Dicer residue S1016, which is required for nuclear accumulation of Dicer. The damage-induced redistribution of a subset of the cellular Dicer pool parallels the Dicer translocation phenotypes observed in *S. pombe* and *C. elegans* upon heat stress (Woolcock et al., 2012) and developmental stimuli (Beshore et al., 2011; Drake et al., 2014), respectively. We further demonstrate phosphorylation of Dicer residues S1728/S1852 promotes the turnover of damage-induced dsRNA. The accumulation of Dicer in damaged nuclei is conserved in mammals. We postulate that the presence of nuclear phosphorylated Dicer promotes the DDR, arguably by processing of damage-induced dsRNA to mediate an RNA-dependent DDR.

### Multiple phosphorylation events regulate nuclear accumulation and activity of human Dicer

Our data suggest that Dicer S1016 phosphorylation may represent a molecular switch that triggers nuclear accumulation. How does S1016 affect Dicer localization? S1016 resides in the platform–PAZ–connector helix cassette, a species-specific sequence that separates the 2-nt 3′-overhang-binding pocket within the PAZ domain and a phosphate-binding pocket within the platform domain (Tian et al., 2014). S1016 residue is conserved between humans and *Drosophila melanogaster* but is altered from serine to asparagine in *C. elegans* (Fig. S4, box). No canonic PAZ domain was identified in *S. pombe* Dcr1. Thus, phosphorylation of the connector helix may have evolved as a regulatory principle for higher eukaryotes to alter Dicer localization or function. The S1016 residue is located at ∼3.2 Å distance to a co-crystallized small RNA substrate and may contribute to dsRNA end recognition (MacRae et al., 2007). The platform–PAZ domain forms a tightly connected, head-like structure in close proximity to the RNase III domains, which are located in the body of the Dicer enzyme (Lau et al., 2012). Thus, S1016 phosphorylation may induce structural rearrangements and thereby also affect the dsRNA binding and processing activity of the dsRBD and RNase III domains in trans. It is tempting to speculate that phosphorylation of S1016 reduces the affinity of human Dicer for its cognate pre-miRNA substrate toward noncognate dsRNA, which is produced in the nucleus and may serve as an anchor to prolong nuclear localization.

The Dicer amino-terminal helicase domain is required for discrimination of dsRNA termini and is supposed to regulate substrate specificities in *C. elegans* and *D. melanogaster* (Welker et al., 2011). Deletion, insertion mutagenesis, or limited proteolysis of the helicase domain impairs dsRNA processing activity of Dicer but not its binding to dsRNA (Zhang et al., 2002; Ma et al., 2008; Soifer et al., 2008). A recently discovered oocyte-specific mouse Dicer isoform Dicer(O), which comprises a truncated amino-terminal helicase domain, shows enhanced processing activity toward long dsRNA substrate during mouse development but no apparent change in subcellular localization (Flemr et al., 2013). The subcellular localization of mouse Dicer has been proposed to be exclusively cytoplasmic Much et al., 2016. Close inspection of mass spectrometry data provided by Much et al. (2016) revealed that several factors involved in RNAPII transcription, such as the RNAPII coactivator p15, the transcriptional coactivator TIF1B, and the pre-mRNA processing factor Fip1, are enriched in HA-Dicer immunoprecipitations, suggesting that a fraction of HA-Dicer interacts with RNA metabolic factors in the nucleus of unperturbed cells. Using these HA::Dicer PMEF cells (Comazzetto et al., 2014) in confocal microscopy and an interspecies heterokaryon assay, we detected nuclear accumulation of HA-Dicer upon induction of DSBs in both mouse and human nuclei.

Localization studies using human Dicer constructs suggest that the helicase domain in the full-length protein occludes the Dicer dsRBD in an auto-inhibitory manner (Doyle et al., 2013). Deletion of the helicase domain or duplication of the dsRBD causes prominent nuclear localization of Dicer. Moreover, a cryptic nuclear localization signal was identified in the dsRBD and partial accumulation of wild-type Dicer was observed upon inhibition of CRM1-dependent nuclear export by LMB. We detected accumulation of damage-induced dsRNA in the absence of Dicer S1728/S1852 phosphorylation. How does phosphorylation of S1728/S1852 promote turnover of dsRNA? The amino-terminal Dicer helicase domain forms a clamp-like structure adjacent to the RNase III active site in the base of the Dicer enzyme (Lau et al., 2012). Phosphorylation of residues S1728/S1852 may cause structural rearrangements that “unfold” the helicase domain, potentially exposing an “unmasked” carboxy-terminal domain for increased dsRNA binding affinity and catalytic activity (Doyle et al., 2013). However, recent data demonstrate that a cytoplasmic amino-terminal deletion mutant of human Dicer efficiently processes exogenous dsRNA substrates in HEK293-derived Dicer knockout cells but fails to accumulate to the nucleus (Kennedy et al., 2015). Collectively, these studies suggest that Dicer is a nuclear-shuttling protein with a relatively short nuclear half live in unperturbed cells. An unmasked carboxy-terminal domain may be necessary but is arguably insufficient for nuclear accumulation of Dicer, which requires additional, damage-induced S1016 phosphorylation.

### p-Dicer processing is linked to DNA repair

Our data suggest that p-Dicer is localized predominantly in damaged nuclei and targeted by PI3K signaling. However, we detect cytoplasmic p-DCR-1 staining when expressing cytoplasmic RFP-Dicer^S1016A^ mutants in the absence of endo-Dicer, indicating that damage-induced signaling can phosphorylate Dicer in the cytoplasm. We noticed that most p-DCR-1 staining in damaged cells is mutually exclusive to total Dicer staining using 13D6 antibody. We detected S1016, but not S1728/1852, Dicer phosphopeptides in samples immunoprecipitated with 13D6 by mass spectrometry. This suggests that Dicer phosphorylation at carboxy-terminal residues S1728/S1852, but not S1016, may mask epitope recognition of 13D6 and that the Dicer signal detected by autoradiography or in ChIP experiments contains S1016, but not S1728/S1852, phosphoresidues. Nevertheless, we detect S1728/S1852 phosphorylated Dicer after immunoprecipitation with H212 or TAP antibodies.

We further show that nuclear Dicer is recruited to DSBs in a dsRNA-dependent manner, suggesting that nascent RNA synthesis is induced at DSBs. Given that recruitment of MDC1 and 53BP1 to DSBs is dependent on both Dicer function and DDRNA (Hawley et al., 2017), we hypothesize that DDRNA may be a product of p-Dicer processing. It is currently unclear how dsRNA is formed upon DNA damage. Intriguingly, DDRNA may also promote changes in chromatin conformation at DSBs through mechanisms that involve Argonaute proteins and recruitment of chromatin-modifying enzymes (Wei et al., 2012; Gao et al., 2014; Wang and Goldstein, 2016). Collectively, these findings suggest that transcription- and p-Dicer-dependent RNA synthesis promote chromatin relaxation at DSBs to generate a “window of opportunity” for recruitment of repair factors engaged in DNA repair.

## Materials and methods

### Tissue culture, cell lines, cloning, and transfection

Mammalian cells were cultured in DMEM (Sigma-Aldrich) with 10% FBS (Thermo Fisher Scientific) at 37°C and 5% CO_2_. Expression of recombinant HEK293T-REx cell lines 293-control_sh (Control KD), 2.B (endo-Dicer knockdown), and 1.3 (endo-Dicer knockdown and Dicer-TAP knock-in; Schmitter et al., 2006) was induced with doxycycline (3 µg/ml; Sigma-Aldrich) for 2–5 d. Wild-type U2OS or *Asi*SI-ER U2OS cells (a gift from the Esashi Laboratory, University of Oxford, Oxford, England, UK) were induced with 4OHT (300 nM; Cayman Chemical) for 2–4 h. Wild-type MEF or PMEF::HA-Dicer PMEF cells (a gift from the O’Carroll Laboratory, Centre for Regenerative Medicine, Edinburgh, Scotland, UK) were cultured at low passages (<20 passages). For site-directed mutagenesis, pTagRFP-Flag-HA-linker-huDicer plasmid (10 ng, a gift from M. Drozdz, Friedrich Miescher Institute for Biomedical Research, Basel, Switzerland), harboring wild-type, RFP-tagged Dicer, was amplified using site-specific primers and Phusion HF high-fidelity polymerase (New England Biolabs, Inc.). For primers, see Table S3. Parental plasmids were digested with 5 U DpnI (Promega) overnight at 37°C, transformed in XL-1-Blue competent cells using heat shock (42°C, 45 s), amplified and purified using the QIAprep spin mini prep kit (QIAGEN). Mutations were confirmed by Sanger sequencing. Transient transfections of HA-tagged *Asi*SI-ER–encoding pBABE plasmid (a gift from the d’Adda di Fagagna Laboratory, Milan, Italy; Iacovoni et al., 2010), shRNA-encoding Dicer knockdown plasmid (Mission shDicer NM_030621; 10271413MN; Sigma-Aldrich), GFP-/RFP-Dicer plasmids (pTagEGFP-Flag-HA-linker-huDicer and pTagRFP-Flag-HA-linker-huDicer, a gift from Maciek Drozdz), or mutants thereof were performed using Lipofectamine 2000 (Invitrogen), polyethylenimine (Sigma-Aldrich), or TransIT-2020 (Mirus Bio) according to the manufacturer’s instructions. siRNA sequences were as follows: siControl (ON-TARGET plus, D-001810-01-05, scrambled sequence; GE Healthcare); siDNA-PKcs, 5′-GGGCGCUAAUCGUACUGAADTDT-3’ (Sigma-Aldrich; a gift from the Gromak Laboratory, Sir William Dunn School of Pathology, Oxford, England, UK).

CRISPR/Cas9 genome editing in human A549 cells was used as described (Ran et al., 2013). A gRNA sequence specifically targeting exon 4 in the *DICER1* gene (5′-CCTTCATAATTTCTCGATAGGGG-3′) was designed and ligated into the hSpCas9-2A-Puro pX459 V2.0 vector (Addgene), expressing Cas9 and puromycin resistance for delivery of the complete CRISPR/Cas9 system. To generate a clonal A549 cell line lacking expression of Dicer (ΔDicer), wild-type A549 cells expanded from single cells were transfected with 10 µg of the CRISPR/Cas9 constructs using the Neon Transfection System (Invitrogen), according to the manufacturer’s instructions. Electroporation settings were as follows: voltage, 1,230 V; pulse width, 30 ms; pulse number, 2; cell density, 5 × 10^6^ cells/ml. Puromycin (1 µg/ml) was added to cells 24 h after transfection. Puromycin selection was performed for a total of 48 h after transfection, refreshing the puromycin media after the initial 24-h treatment. Puromycin-resistant cells were grown to confluency and clonally selected. PCR with locus-specific primers (forward, 5′-CAAAAAGGCTCAATTAGATACACT-3′; reverse, 5′-ATAATATGGCTGTGGGGATCT-3′) was used to amplify a 650-bp region around the CRISPR target site and to verify mutation of the *DICER1* gene. TIDE analysis was performed using the TIDE Software online webtool (http://tidecalculator.nki.nl/).

### Chemicals and antibodies

Cells were treated with the following chemicals: DMSO (0.1%, Control; Sigma-Aldrich), STS (3 µM; LKT Labs), Eto (25 µM; Sigma-Aldrich), H_2_O_2_ (500 µM; Sigma-Aldrich), phleomycin (5 mg/ml; Cayman Chemical), MMS (500 µM; Sigma-Aldrich), HU (2 mM; Sigma-Aldrich), LMB (5 nM; Cayman Chemical), ATM inhibitor KU-55933 (5 µM; Sigma-Aldrich), ATR inhibitor VE-821 (1 µM; Sigma-Aldrich), and PI3K inhibitor LY294002 (5 µM, New England Biolabs, Inc.) and osmotic stress (0.1× or 10× PBS) for 2 h, unless stated differently. Cells were exposed to γ-irradiation for up to 10 min, equivalent to doses up to 10 gy.

The following primary antibodies were used: anti-Dicer (13D6, ab14601, mouse; Abcam); anti-Dicer (A-2, sc-136891, mouse; Santa Cruz Biotechnology, Inc.) and anti-Dicer (H212, sc-30226, rabbit; Santa Cruz Biotechnology, Inc.); anti-p-DCR-1 (gift from S. Arur's laboratory, MD Anderson Cancer Center, Houston, TX; Drake et al., 2014); anti–α-tubulin (YL1/2, ab6160, rat; Abcam); anti-γH2A.X (S139, 05–636, mouse; EMD Millipore); anti-GFP-tag (GT859, GTX628528, mouse; GeneTex Inc.); anti-RFP-tag (RF5R, MA5-15257, mouse; Thermo Fisher Scientific); anti-Rad21 (05–908, mouse; EMD Millipore); anti-J2 (10010200, mouse; SCICONS); anti-ATM (2C7, sc-23921, mouse; Santa Cruz Biotechnology, Inc.), anti-pATM (S1981, ab81292, rabbit; Abcam), and anti-53BP1 (H-300, sc-22760, rabbit; Santa Cruz Biotechnology, Inc.); anti-Grp75 (JG1, ab2799, mouse; Abcam), and anti-histone H3 (ab1791, rabbit; Abcam); anti-HA (3F10, rat; Roche); anti-pATM/ATR substrates mix (SxQ, D23H2/D69H5, 9670, rabbit), anti-cleaved PARP (5625, rabbit), anti-pChk1 (S345, 133D3, rabbit), and anti-pChk1 (S317, D12H3, rabbit; Cell Signaling Technology); anti-Ki-67 (SP6, ab16667, rabbit; Abcam), and anti-DNA-PKcs (18–2, ab1832, mouse; Abcam); and anti-TAP human IgG sepharose 6 FastFlow beads (17–0969-01; Invitrogen).

### Proliferation assay

Proliferation of HEK293 cells was measured by electric impedance detection using the xCELLigence device (ACEA Biosciences Inc). 2 × 10^3^ HEK293 cells were plated on an electronic plate, which was capable of measuring electric impedance in real time by electrodes in direct contact with adherent cells. Impedance increases with the area on electrodes, which is covered by proliferating cells and is termed cell index. Cells were cultured at 37°C and 5% CO_2_, and impedance was measured in 15-min intervals in triplicates.

### Subcellular fractionation, whole cell lysis, and co-immunoprecipitation

Subcellular fractionation was performed as previously described (Redfern et al., 2013). HEK293 cells were lysed in five volumes of hypotonic lysis buffer (10 mM Hepes, pH 7.9, 60 mM KCl, 1.5 mM MgCl_2_, 1 mM EDTA, 1 mM DTT, 0.075% NP-40, and 1× protease/phosphatase inhibitor cocktails; Roche) and were incubated for 10 min at 4°C with rotation. Nuclei were pelleted by centrifugation (1,200 rpm; 4°C) for 10 min. The cytoplasm was collected from the supernatant. Nuclei were washed five times in 800 µl hypotonic lysis buffer without NP-40 and lysed in 1 volume of nuclear lysis buffer (20 mM Hepes, pH 7.9, 400 mM NaCl, 1.5 mM MgCl_2_, 0.2 mM EDTA, 1 mM DTT, 5% glycerol, and 1× protease/phosphatase inhibitor cocktails; Roche). Lysates were diluted with two volumes dilution buffer (20 mM Hepes, pH 7.9, 1.6% Triton X-100, 0.2% sodium deoxycholate, and 1× protease/phosphatase inhibitor cocktails; Roche), followed by 10 s sonication with a Bioruptor (Diagenode) at low energy and incubation with 10 U benzonase (Sigma-Aldrich) for 5 min. Lysates were centrifuged (13,500 rpm; 4°C, 10 min), and the supernatant was collected as a soluble nuclear fraction. 10% of subcellular fractions were boiled in 0.25× volume of 4× SDS-PAGE sample buffer (12% SDS, 40 mM Tris HCl, pH 7.4, 40% glycerol, 3% β-mercaptoethanol, and 1% bromophenol blue) at 95°C for 5 min, sonicated, and analyzed by Western blot using precast gels (Mini-PROTEAN TGX; Bio-Rad Laboratories). Whole cell extracts (WCEs) were lysed directly in 4× SDS-PAGE sample buffer and stained with Ponceau *S* (Sigma-Aldrich) before antibody hybridization. Signals were quantified using ImageJ (National Institutes of Health).

Purified, subcellular fractions and whole cell lysates were precleared with protein A/G agarose beads (EMD Millipore) for 30 min. Samples were incubated with 5 μg primary antibodies for 2 h and pulled down using protein A/G agarose beads for 45 min. For TAP-IP, precleared samples were incubated with IgG sepharose beads (Invitrogen) for 90 min. IP samples were washed three times for 10 min with WCE lysis buffer (20 mM Tris, pH 7.5, 150 mM NaCl, 0.1% NP-40, 2 mM MgCl_2_, 50 mM NaF, and 1× protease/phosphatase inhibitor cocktails; Roche), eluted with SDS-PAGE sample buffer and analyzed by Western blot using standard or Phos-tag–containing SDS-PAGE gels (Wako Pure Chemical Industries). For Phos-tag analysis, samples were separated for 12 h at 4°C and Dicer migration was visualized by immunoblotting. Gels were washed in transfer buffer containing 10 mM EDTA for 10 min before protein transfer. Signals were quantified using AIDA. Distances were measured in migration units relative to wells.

### [^32^P]Orthophosphate metabolic labeling

In vivo metabolic labeling was performed as previously described (Burger and Eick, 2016). HEK293 cells were depleted from the endogenous phosphate pool by preculture in OptiMEM (Gibco) for 2 h. 15 µCi/ml [^32^P]orthophosphate (3,000 Ci/mM; PerkinElmer) and DNA-damaging agents were added simultaneously and incubated for an additional 2 h. Dicer was immunoprecipitated from whole cell lysates. De novo phosphorylation was analyzed by autoradiography after calf intestine phosphatase (CIP; Invitrogen) treatment using 1 U for 1 h at 37°C. Upon separation by SDS-PAGE, signals were visualized by autoradiography and quantified using a Phosphorimager (Fujifilm) and AIDA software. CIP was visualized using a silver staining kit (Invitrogen), according to the manufacturer’s protocol.

### Immunofluorescence microscopy and heterokaryon formation

*Asi*SI-ER U2OS or HEK293 cells were washed in 1× PBS, fixed on coverslips with 3% PFA in PBS for 10 min, washed and incubated with 50 mM ammonium chloride in PBS for 10 min, washed in PBS, permeabilized with PBS/0.1% Tween for 7 min, and blocked with PBS/10% FBS for 2 h at 4°C. Primary antibodies were incubated overnight at 4°C in PBS/0.15% FBS. Cells were washed in PBS/0.1% Triton X-100 (3 min, three times). Alexa Flour 488–, 555–, or 647–conjugated secondary antibodies (Invitrogen) were incubated in PBS/0.15% FBS at RT for 2 h in a humidified chamber. Cells were washed in PBS/0.1% Triton X-100 (3 min, three times). Nuclei were counterstained and mounted with DAPI-containing Mowiol (EMD Millipore). Samples were imaged by epifluorescence and confocal microscopy (BX61 and FV1000; Olympus) using equal exposure times. For epifluorescence microscopy, samples with 1.5-thick coverslips were imaged using a 60× 1.35 NA oil immersion objective lens and a CoolSNAP HQ2 camera (Roper Technologies). Image Z stacks, comprising 12 images, 0.2 µm apart, were collected and maximum projected to give a single image for each color channel.

For confocal imaging, samples with 1.5-thick coverslips were imaged using a an FV1000 confocal system on an Olympus IX-81 microscope with photomultiplier tube detectors and Olympus PlanApo N, 60×/1.35NA lens at RT. DAP)-containing Mowiol (EMD Millipore) was used as the imaging medium. DAPI; Alexa Fluor 488, 539, and 635 (Thermo Fisher Scientific); RFP; and eGFP channels were used for acquisition with Olympus Fluoview software. ImageJ software (Schindelin et al., 2012) was used for further processing of the images. For quantitation of γH2A.X-positive cells, >200 wild-type and Dicer KD cells were counted and scored as γH2A.X-positive, if they comprised five or more γH2A.X spots. For RFP-Dicer wild-type and mutants, >50 transfected cells were counted for each construct and analyzed with ImageJ software. For dsRNA, >50 cells from each sample were analyzed with ImageJ software. Co-localization was quantified with an RGB-profiler (ImageJ). All quantifications represent several cells that have shown phenotype or percentage of positive cells (see figure legends for details; *n*, number of cells).

For heterokaryon formation, wild-type or recombinant MEF cells expressing wild-type or endogenously tagged HA-Dicer (PMEF::HA-Dicer) were grown to 70–80% confluency. *Asi*SI-ER U2OS cells were seeded on top of the MEF layer before membrane fusion. Mixed-cell populations were grown in the presence of cycloheximide (50 µg/ml) for 4 h before fusion. For heterokaryon formation, cells were washed with warm 1× PBS, incubated with 100 µl warm PEG-3000 solution (50% wt/vol in PBS) for 2 min, and washed with 1× PBS five times. Heterokaryons were cultured for 4 h in cycloheximide-containing medium in the presence or absence of 4OHT before fixation. Alexa Fluor 647 phalloidin (Thermo Fisher Scientific) was used to stain the cytoskeleton.

### ChIP

ChIP analysis was performed as previously described (Neve et al., 2016). *Asi*SI-ER U2OS cells were fixed with 1% formaldehyde (10 min, 37°C). Formaldehyde was inactivated by the addition of glycine to a final concentration of 0.125 M (10 min, 37°C). Cells were washed twice with 5 ml ice-cold PBS and then scraped into 15-ml tubes. Samples were centrifuged for 5 min at 1,600 rpm at 4°C. Cells were resuspended in 500 µl of cell lysis buffer (5 mM Pipes, pH 8.0, 85 mM KCl, 0.5% Nonidet P-40, 1 mM PMSF, 1 µg/ml pepstatin A, 1 µg/ml leupeptin, and 1× protease/phosphatase inhibitor cocktails; Roche) and incubated on ice for 10 min. Nuclei were collected by centrifugation for 5 min at 3,000 rpm at 4°C and were resuspended in 400 µl ice-cold nuclear lysis buffer (1% SDS, 10 mM EDTA, 50 mM Tris-HCl, pH 8.0, 0.5 mM PMSF, 0.8 µg/ml pepstatin A, 1 µg/ml leupeptin, and 1× protease/phosphatase inhibitor cocktails; Roche) and were then incubated on ice for 10 min. Samples were sonicated to an mean length of 300–500 bp, kept on ice (30 s sonication and 30 s rest) and spun for 10 min at 13,000 rpm at 4°C to remove cell debris. The supernatant was diluted by the addition of 2.5 volumes IP dilution buffer (0.01% SDS, 1.1% Triton X-100, 1.2 mM EDTA, 16.7 mM Tris-HCl, pH 8.0, 167 mM NaCl, 0.5 mM PMSF, 0.8 µg/ml pepstatin A, 1 µg/ml leupeptin, and 1× protease/phosphatase inhibitor cocktails; Roche). Diluted ChIP samples were precleared by incubation with protein A/G agarose beads (EMD Millipore) or magnetic beads (Invitrogen) for 30 min and aliquoted into various IP samples. RNA digestions were performed using RNase III (1 U; New England Biolabs, Inc.) for 1 h at 37°C. Specific antibodies (5 µg/100 µg chromatin) were added to samples and incubated overnight at 4°C on a rotating wheel. Immune complexes were pulled down at 4°C with 40 µl of protein A/G agarose beads or magnetic beads for 1 h and washed with buffers A–D: A, 0.1% SDS, 1% Triton X-100, 2 mM EDTA, 20 mM Tris-HCl, pH 8.0, and 150 mM NaCl; B, 0.1% SDS, 1% Triton X-100, 2 mM EDTA, 20 mM Tris-HCl, pH 8.0, and 500 mM NaCl; C, 0.25 M LiCl, 1% NP-40, 1% sodium deoxycholate, 1 mM EDTA, and 10 mM Tris-HCl, pH 8.0; and D, 10:1 TE buffer, pH 8.0. Immune complexes were eluted with 500 µl IP elution buffer (1% SDS, 0.1 M NaHCO_3_) for 30 min on a rotating wheel. Reversal of cross-links was performed by adding 0.3 M NaCl, 3 µg/ml RNase A, 10 µl of 0.5 M EDTA, 20 µl of 1 M Tris-HCl, pH 6.5, and 2 µl of 10 mg/ml proteinase K; then, incubating at 65°C overnight. DNA was purified by phenol/chloroform extraction and recovered in distilled H_2_O. Signals represent the mean of at least three biological repeats expressed as the percentage of input, as ratios, or as fold-change relative to controls. For primers see Table S4.

### Genomics and bioinformatics analysis

Genomics and bioinformatics analysis ChIP-seq (White et al., 2014) data were mapped with Bowtie 2 (version 2.2.5) after trimming of the first poor-quality nucleotide with Cutadapt (version 1.8.3). Duplicate reads were removed with Samtools (version 1.1).

HEK293 Dicer ChIP-Seq data were taken from White et al. (2014). Adapter and contaminating sequences were identified with fastQC (version 0.11.5; Available online at**:** http://www.bioinformatics.babraham.ac.uk/projects/fastqc; Babraham Bioinformatics) and were trimmed in single-end mode using Cutadapt (version 1.8.3). These sequences include 5′-AGATCGGAAGAGCTCGTATGCCGTCTTCTGCTTG-3′, 5′-TCGTATGCCGTCTTCTG-3′, and 5′-CTGTAGGCACCATCAAT-3′. Only reads of more than 10 nt were kept and mapped with Bowtie 2 (version 2.2.5). Duplicate reads were removed with Samtools (version 0.1.19). Coverage bigWig graphs were computed with deepTools 2 bamCoverage. The profile around the *Asi*SI sites was computed with deepTools 2 computeMatrix reference-point and normalizing to the library read count.

Data were visualized with ggplot2 (http://www.ggplot2.org/) in R software (http://www.R-project.org/) by applying a 1,000-nt rolling mean to the trimmed signal mean (2% of most-extreme values trimmed from both ends). The rolling mean was computed with the roll_mean function, and the rolling SD was computed with the roll_sd function from the RcppRoll package.

We used γH2A.X and H2A.X ChIP-seq data from Yata et al. (2014). The log_2_ ratio of γH2A.X/H2A.X was computed in 10-kb bins with deepTools 2 bamCompare, with read count normalization. From this ratio, peaks were called with a custom script by using MATLAB (http://www.mathworks.co.uk/matlabcentral/fileexchange/25500-peakfinder; MathWorks). Using Perl programming language, peaks were extended to either side until at least eight bins had ≤0 signal. Peaks <40 kb long were discarded. *Asi*SI sites overlapping those peaks were ranked according to the log_2_ (γH2A.X/H2A.X) signal in a (*Asi*SI −25 kb, *Asi*SI +25 kb) window. *Asi*SI sites <10 kb apart were summarized into the one with the highest log_2_ (γH2A.X/H2A.X) in the 50-kb window. The top 200 of these *Asi*SI sites were considered as efficiently cut upon damage induction. The remaining *Asi*SI sites were also ranked according to log_2_ (γH2A.X/H2A.X) signal in the 50-kb window. 200 *Asi*SI sites within 500 nt of a gene (RefSeq V9 – hg38) with the lowest log_2_(γH2A.X/H2A.X) signal were considered as not cut upon damage induction to serve as the negative control. For Dicer signal ratio box plots between induced and noninduced cells at γH2A.X-positive or γH2A.X-negative sites (Fig. S2 D), we used 99 cut *Asi*SI sites, as annotated in Aymard et al. (2014). The ratio was computed via deepTools 2 bamCompare with read count normalization.

Code description is as follows: (a) peakf.m: MATLAB code to find peaks in 1-kb γH2A.X/H2A.X data (uses publicly available peakfinder.m code http://uk.mathworks.com/matlabcentral/fileexchange/25500-peakfinder-x0-sel-thresh-extrema-includeendpoints-interpolate-); (b) peak_matlab.pl: Perl code to further process, summarize, and exclude peaks found by peakf.m code; (c) *Asi*SI_gamma_signal.pl: Perl code to compute γH2A.X/H2A.X cumulative signal in the peaks output by peak_matlab.pl; (d) Dicer_signal_atsites.pl: Perl code that reads the deepTools output matrix and computes signal sum within ±500 nt of provided γH2A.X+/γH2A.X− *Asi*SI sites; and (e) box_plot_figures.R: R code to plot metagene profiles from deepTools output matrix and box plots for Dicer_signal_atsites.pl output.

### Mass spectrometry

For mass spectrometry analysis, SDS-PAGE–purified IP samples were digested in the gel with trypsin. Peptides were analyzed on a nano ultra-HPLC system coupled to a QExactive mass spectrometer (Thermo Fisher Scientific). Phosphopeptides were purified by C18 reverse-phase chromatography and were enriched using titanium-dioxide columns before analysis.

In detail, endo-Dicer was purified form subcellular fractions of HEK293 cells. Samples were separated by SDS-PAGE and cut in gel slices. For in-gel tryptic digestion, slices were briefly washed with 50% ACN and dried in 100% ACN at 37°C for 10 min. Dried slices were incubated with 2% Tris (2-carboxyethyl) phosphine diluted in 100 mM tetraethylammonium bromide (TEAB) at RT for 30 min. Tris (2-carboxyethyl) phosphine was removed, and slices were incubated in 50 mM 2-chloracetamide, diluted in 100 mM TEAB in the dark at RT for 30 min. 2-Chloracetamide was removed, and slices were dried in 100% ACN at 37°C for 10 min. ACN was removed, and trypsin (500 ng/IP), diluted in 50 mM TEAB, was added. Slices were digested at 37°C overnight. Supernatants were collected and reduced to small volumes on a speedvac for several hours. Peptides were loaded on C18 columns. Columns were sequentially equilibrated with 100% ACN and 0.1% trifluoroacetic acid (TFA). Peptides were loaded and washed with 0.1% TFA. Peptides were sequentially eluted with 50% ACN and 0.1% TFA, transferred to glass vials, and dried on a speedvac.

Peptides were resuspended in 5% formic acid and 5% DMSO and then trapped on a C18 PepMap100 precolumn (300 µm inner diameter × 5 mm, 100 Å; Thermo Fisher Scientific) using 0.1% formic acid in water at a pressure of 500 bars and analyzed on an Ultimate 3000 ultra-HPLC system (Thermo Fisher Scientific) coupled to a QExactive mass spectrometer (Thermo Fisher Scientific). The peptides were separated on an in-house packed analytic column (360 µm × 75 µm inner diameter packed with ReproSil-Pur 120 C18-AQ, 1.9 µm, 120 Å; Dr. Maisch GmbH) and then electrosprayed directly into an QExactive mass spectrometer (Thermo Fisher Scientific) through an EASY-Spray nano-electrospray ion source (Thermo Fisher Scientific) using a linear gradient (length: 60 min, 7–28% solvent B [0.1% formic acid in ACN], flow rate: 200 nl/min). Raw data were acquired on the mass spectrometer in a data-dependent mode. Full-scan, mass spectra were acquired in the Orbitrap (scan range 350–2000 *m/z*, resolution 70,000, AGC target 3 × 10^6^, maximum injection time 100 ms). After mass spectrum scans, the 20 most-intense peaks were selected for higher-energy collisional dissociation fragmentation at 30% of normalized collision energy. The higher-energy collisional dissociation spectra were also acquired in the Orbitrap (resolution 17,500, AGC target 5 × 10^4^, maximum injection time, 120 ms) with first-fixed mass at 180 *m/z*.

Generated raw data files were processed using MaxQuant (version 1.5.0.35; Max Planck Institute of Biochemistry), integrated with the Andromeda search engine, as previously described (Cox and Mann, 2008; Cox et al., 2011). To identify protein groups, peak lists were searched against human database (Swiss Prot, version 04/13) as well as a list of common contaminants by Andromeda. Trypsin with a maximum number of missed cleavages of 2 was chosen. Acetylation (protein N-term, i.e., only the amino terminus of the protein), oxidation (M), and phosphorylation (S, T, and Y) were used as variable modifications, whereas carbamidomethylation (C) was set as a fixed modification. A protein and posttranslational modification false-discovery rate of 0.01, a minimum score of 40, and a localization probability of >0.7 for phosphopeptides were set. Match between runs was applied.

Initial protein-level data processing was performed using R software. Protein-intensity values from MaxQuant were normalized by log transformation, median centered, and scaled by median absolute deviation. Proteins for which neither condition had two nonmissing values were discarded. For the remaining proteins, missing values were imputed by two strategies. For proteins missing only one value from a condition, the missing value was imputed by random draw from a normal distribution with the mean equal to the nonmissing value from the same condition, and SD equal to the SD of the two values from the other condition. For the remaining proteins, for which both values were missing from a condition, the missing values were assumed to be due to left-censoring (because of below-detection limit abundance), and replacements were input by the QRILC method (random draws from a truncated distribution with parameters estimated using quantile regression from the distribution of all values in that condition) using the imputeLCMD package. After missing-value imputation, each condition was recentered and rescaled, and p-values were calculated using a two-tailed, paired *t* test assuming equal variance. False discovery rate–adjusted p-values (q values) were calculated using the q value package and the bootstrap method to estimate pi0.

### Online supplemental material

Supplemental material contains five figures. Fig. S1 displays damage-induced phosphorylation of Dicer. Fig. S2 demonstrates Dicer chromatin occupancy. Fig. S3 depicts the relevance of DNA damage signaling for Dicer phosphorylation. Fig. S4 shows detection of damage-induced phosphopeptide, and Fig. S5 shows additional controls and quantification. Supplemental material also contains four tables with peptides and primers and five source code files.

## Supplementary Material

Supplemental Materials (PDF)
