# Supplementary material for: Nuclear phosphorylated Dicer processes double-stranded RNA in response to DNA damage
Source: J Cell Biol. 2017 Aug 7;216(8):2373–89. doi: 10.1083/jcb.201612131 (PMC5551710; doi:10.1083/jcb.201612131)
Supplement: Supplemental Materials (PDF) [file JCB_201612131_sm.pdf]

# Supplemental material

JCB

Burger et al., https://doi.org/10.1083/jcb.201612131

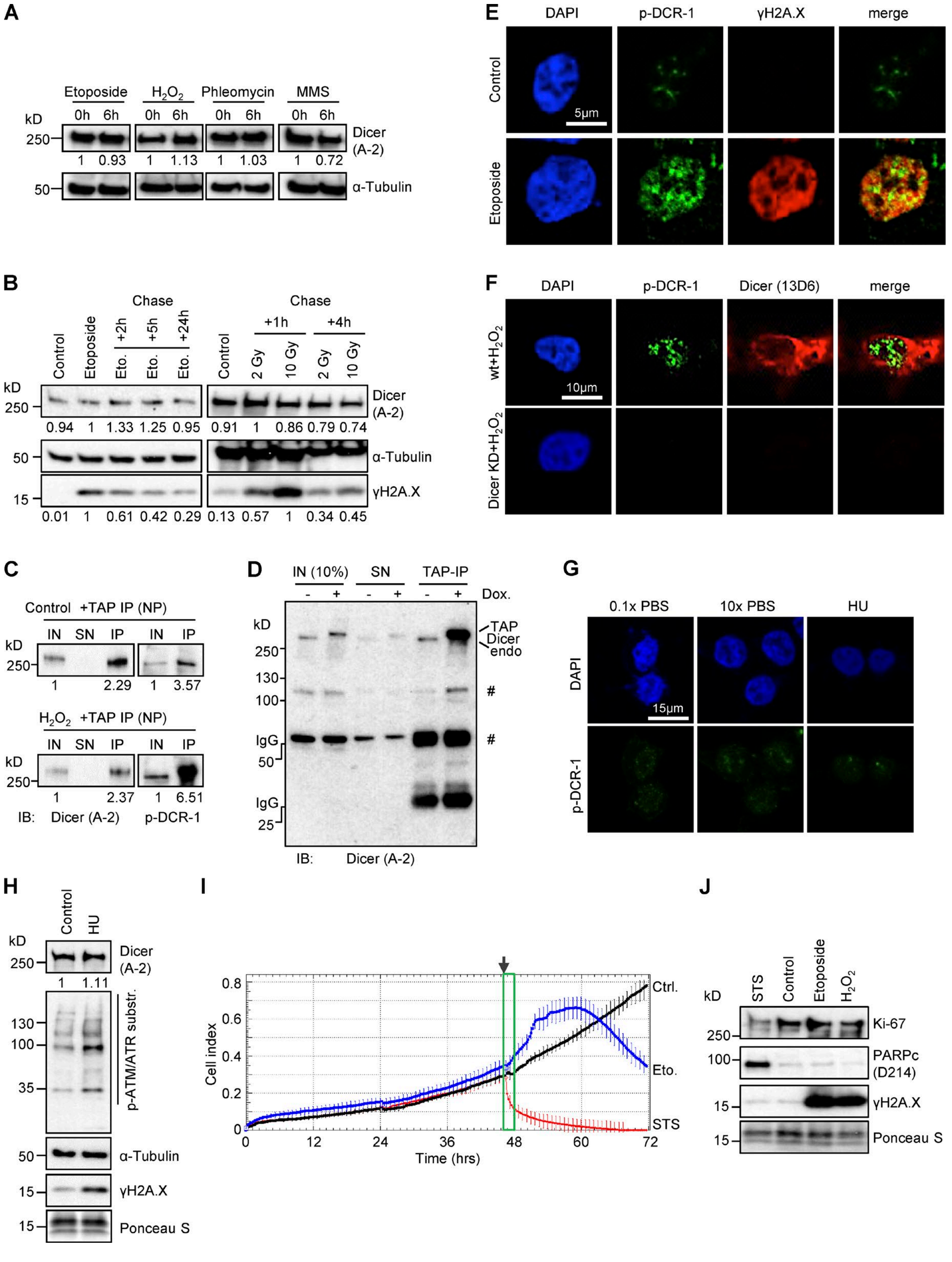


Figure S1. **Expression and subcellular localization of Dicer upon DNA damage in HEK 293 cells.** (A and B) Immunoblots detecting total Dicer (A-2) and phosphorylated histone variant H2A.X (γH2A.X, S139) after continuous DNA damage (A) or pulse-chase treatment (B). Immunoblots were quantified using ImageJ. (C and D) Immunoblots detecting total (A-2) or phosphorylated (p-DCR-1) recombinant TAP-tagged Dicer after depletion of endogenous Dicer, reexpression of TAP-tagged Dicer, and immunoprecipitation from nondamaged (control) and damaged ($H_2O_2$) nuclear fractions. Immunoblots were quantified using ImageJ. #, nonspecific signal. (E) Confocal imaging of phosphorylated Dicer (p-DCR-1) and γH2A.X. (F) Confocal imaging of phosphorylated (p-DCR-1) and total (13D6) Dicer in wild-type and Dicer-depleted (Dicer KD) cells. (G) Confocal imaging of phosphorylated Dicer (p-DCR-1) in HEK293 cells. HU, hydroxyurea. (H) Immunoblots detecting total Dicer (A-2), phosphorylated ATM/ATR substrates and γH2A.X in HEK293 cells. α-Tubulin, loading control. (I) Real-time proliferation assay measuring the "cell index" as a function of impedance change over time. Arrowhead, addition of drugs; green box, time frame of analysis. (J) Immunoblots detecting Ki-67, cleaved Poly ADP-ribose polymerase (PARPc) and γH2A.X after drug treatment. $H_2O_2$, hydrogen peroxide; MMS, methyl methanesulfonate; STS, staurosporine; Gy, gray; IN, input; SN, supernatant; IP, immunoprecipitation; IB, immunoblot.

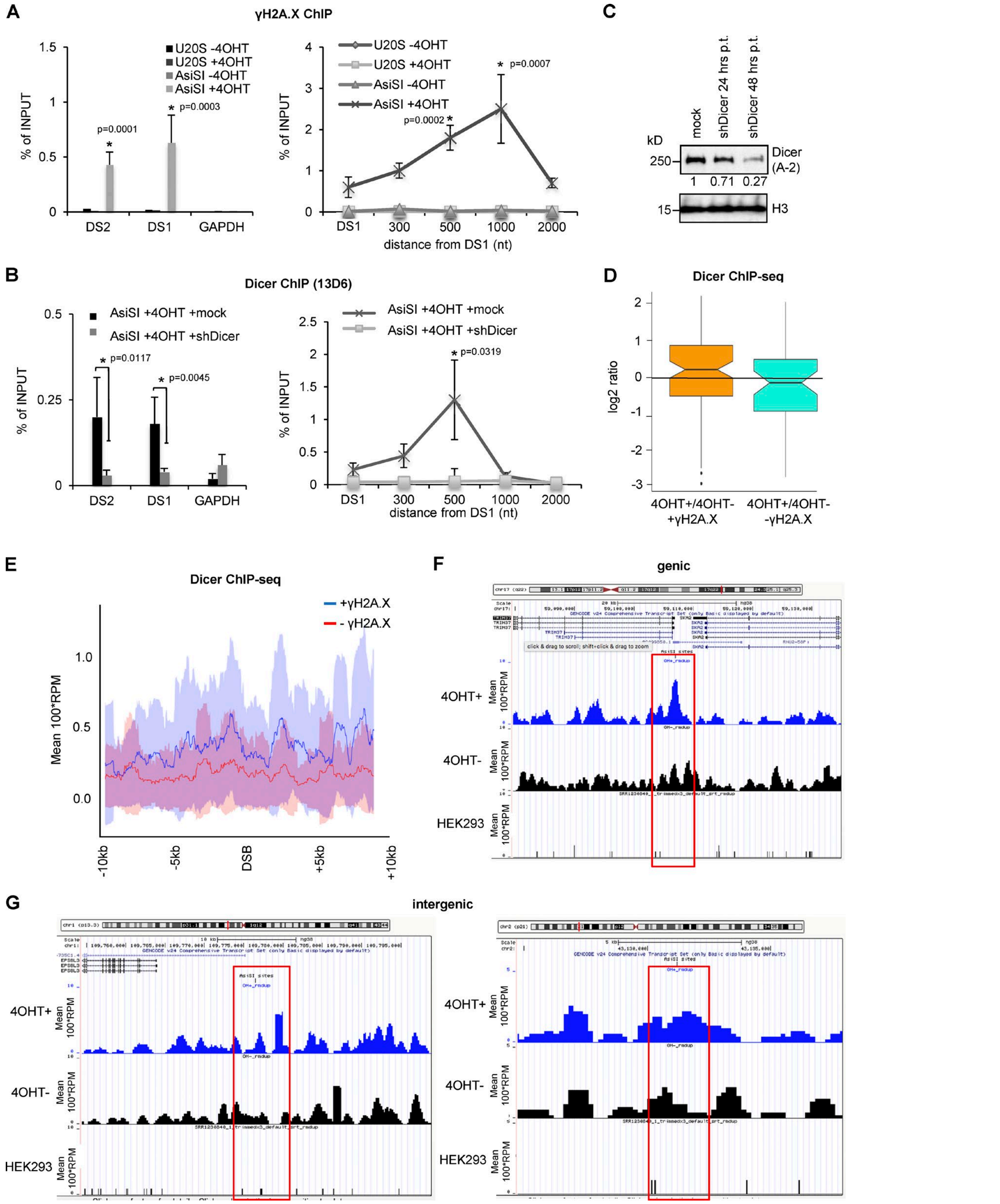


Figure S2. **Dicer occupancy at DNA double-strand breaks in *AsiSI*-ER U2OS cells.** (A and B) ChIP analysis showing γH2A.X (A) and Dicer (B) occupancy at DSBs DS1/2 in wild-type and Dicer-depleted *AsiSI*-ER U2OS cells using site-specific primers. GAPDH, control locus. 4OHT, 4-hydroxytamoxifen; *, $P < 0.05$; error bars, means ± SEM of three biological replicates. (C) Immunoblots detecting Dicer levels after transfection of Dicer-targeting shDicer. p.t., posttransfection. Control, mock-transfected cells. (D) Box plot showing levels of Dicer ChIP-seq in $\log_2$ ratios of $4OHT^+/4OHT^-$ conditions for both, 99 γH2A.X-positive and 99 γH2A.X-negative sites in −500 bp/+500 bp around the *AsiSI* site window. Horizontal black line extends the *y*-axis 0 value for better visibility. Median value is indicated as a horizontal black line within the box plots. $P = 0.0551$, Wilcoxon rank-sum test. (E) ChIP-seq signal in HEK293 cells at γH2A.X-positive *AsiSI* sites (top 200, blue) and genic γH2A.X-negative *AsiSI* sites (bottom 200, red) after duplicate read removal. A rolling mean of 1 kb was applied after removal of 2% of the top and bottom values. Shadow, rolling SD. (F and G) Snapshots showing examples of Dicer binding at *AsiSI* target sites before ($4OHT^-$) and after ($4OHT^+$) DNA damage at genic and intergenic cut *AsiSI* sites. Red box, proximal region to *AsiSI* site.

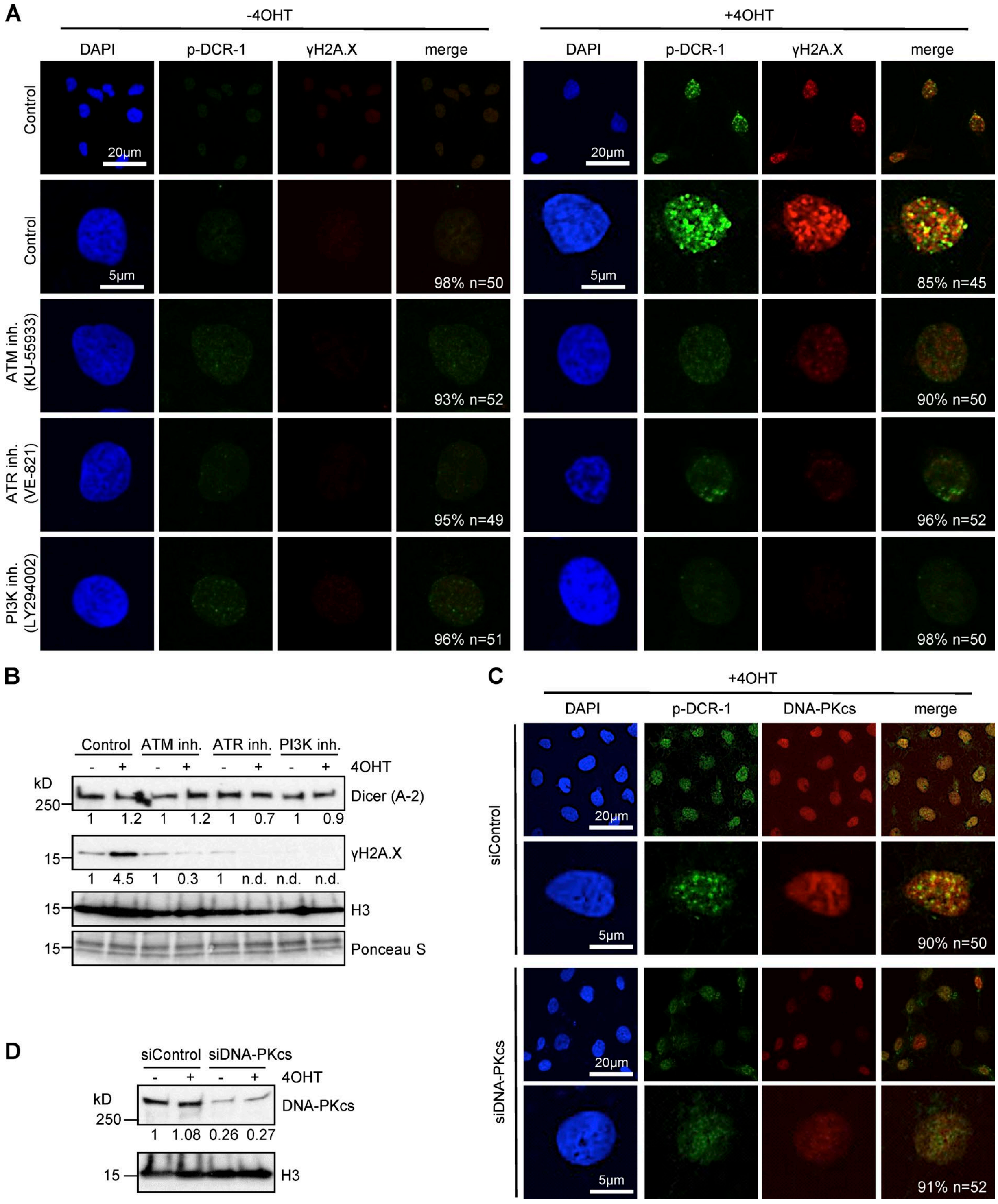


Figure S3. **Phosphorylation of Dicer by phosphatidylinositol-3-kinase (PI3K) signaling in *Asi*SI-ER U2OS cells.** (A) Confocal imaging of phosphorylated Dicer (p-DCR-1) and γH2A.X after preincubation with kinase inhibitors. All quantifications represent the number of cells that exhibit the shown phenotype. (B) Immunoblots detecting γH2A.X levels after preincubation with kinase inhibitors. n.d., not detectable. (C) Confocal imaging of phosphorylated Dicer (p-DCR-1) and γH2A.X after depletion of DNA-dependent protein kinase, catalytic subunit (DNA-PKcs). All quantifications represent the number of cells that have the shown phenotype. In siDNA-PKcs cells, only cells with efficient depletion of DNA-PKcs were considered for quantification. (D) Immunoblots detecting DNA-PKcs after depletion of DNA-PKcs by siRNA.

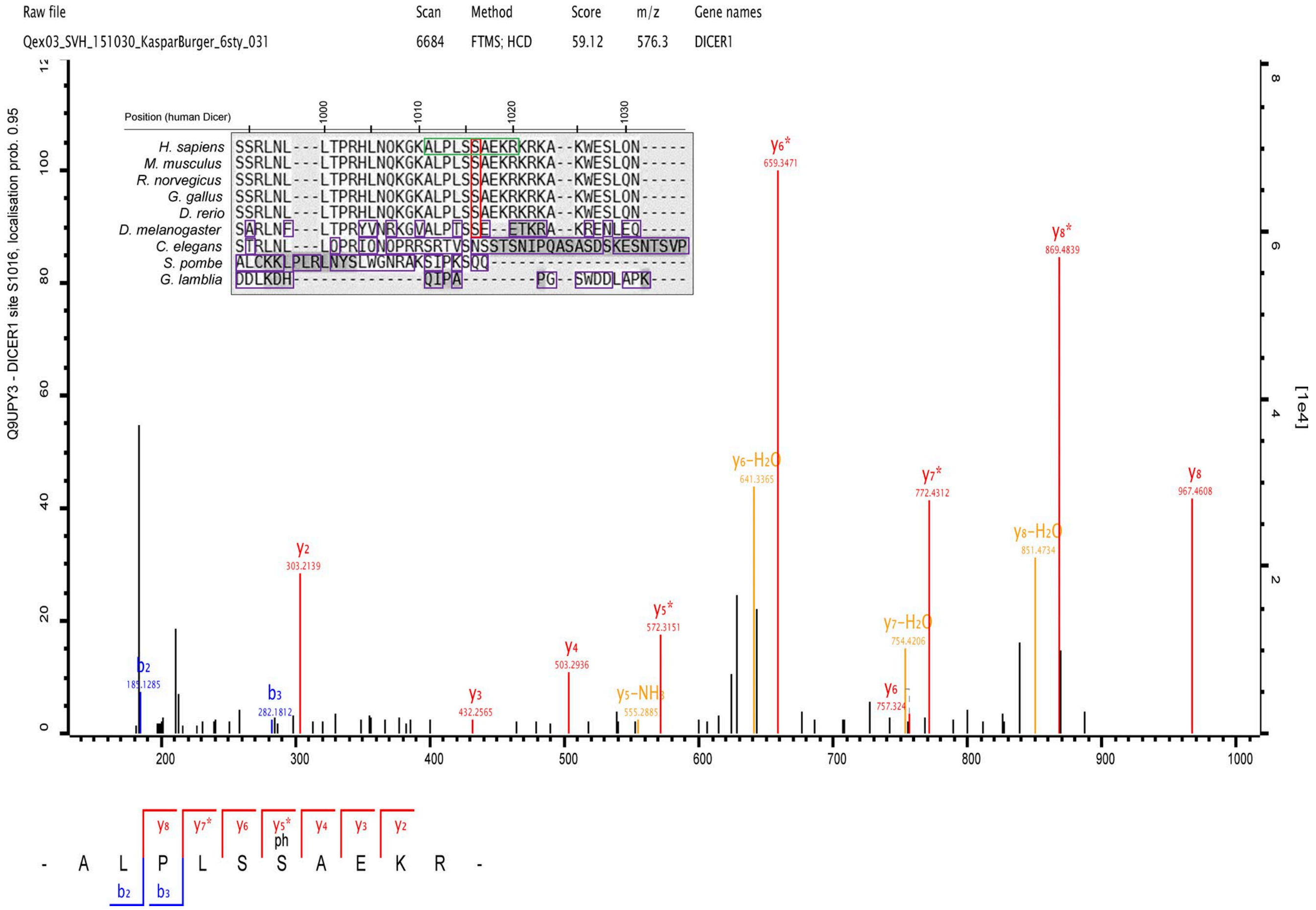


Figure S4. **Chromatogram of Dicer spectral counts for residue S1016 in HEK293 in cells.** Fragmentation spectra of ion series at Dicer residue S1016 in HEK293 cells. Box, alignment of the platform–PAZ–connector helix cassette aa sequence surrounding residue S1016 across species; green, damage-induced phosphopeptide; red, conserved S1016 residue; purple, nonconserved residues. Fragmentation spectra of individual phosphopeptides available on request.

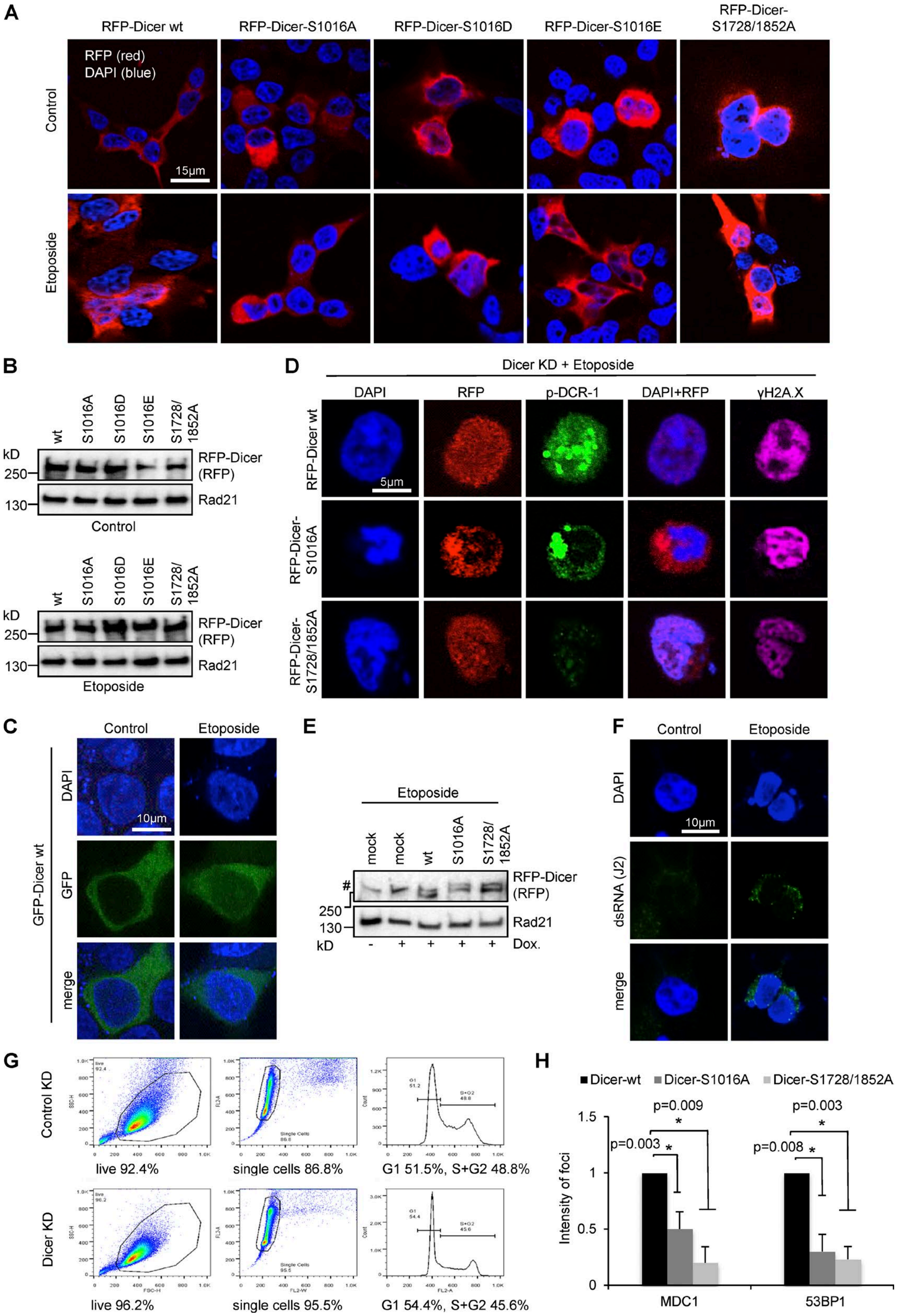


Figure S5. **Differential subcellular localization of RFP-Dicer mutants in HEK293 cells.** (A) Confocal imaging of RFP-Dicer constructs. Merged images are shown. (B) Immunoblots detecting expression of RFP-Dicer constructs. Rad21, loading control. (C) Confocal imaging of GFP-tagged wild-type Dicer, $n > 30$. (D) Confocal imaging of RFP-Dicer constructs, phosphorylated Dicer (p-DCR-1), and γH2A.X in Dicer-depleted (Dicer KD) cells transfected with RFP-Dicer constructs, $n > 30$. (E) Immunoblots detecting expression of RFP-Dicer constructs in wild-type and Dicer-depleted (Dicer KD) cells. (F) Confocal imaging of dsRNA (J2), $n > 30$. (G) Flow cytometry analysis assessing the cell cycle distribution of Dicer KD cells. Control KD, induction of scrambled shRNA knockdown. Dox., doxycycline; Rad21, loading control; #, unspecific signal. (H) Quantification of MDC1 and 53BP1 foci per cell, as shown in Fig. 7 (E and F) in 4OHT$^{+}$ 2 h condition. Error bars are derived from three biological repeats. At least 40 cells were counted per repeat. *, $P < 0.05$.

Table S1. **Quantification of damage-induced nuclear Dicer phosphoresidue serine-1016**

| Dicer peptide sequence | Modification | Mass | PEP | Score | Intensity control | Intensity etoposide | Ratio Eto:Ctrl |
|---|---|---|---|---|---|---|---|
| ALPLSSAEKR | Phospho (STY) | 1,150.575 | 0.004008 | 53.03 | 195,960 | 654,860 | 3.342 |
| APKEEADYEDDFLEYDQEHIR | — | 2,611.135 | $2.62^{-8}$ | 67.93 | 303,760 | 242,380 | 0.798 |
| EAGKQDPELAYISSNFITGHGIGK | — | 2,531.266 | $9.83^{-20}$ | 92.79 | $3^{6}$ | 2,750,100 | 0.912 |
| RSEEDEEKEEDIEVPK | — | 1,959.891 | 0.001267 | 41.54 | 249,080 | 282,200 | 1.133 |

PEP, posterior error probability.

Table S2. **Nuclear Dicer phosphopeptides identified by mass spectrometry**

| Residue | Peptide |
|---|---|
| S1016 | ALPLSpSAEKR |
| S1142 | TSpSLENHDQMSVNCR |
| S1160 | TLLSEpSPGK |
| S1250 | pSTSDGSPVMAVMPGTTDTIQVLK |
| S1468 | KIpSLSPFSTTDSAYEWK |
| S1470 | ISLpSPFSTTDSAYEWK |
| S1868 | ELLEMEPETAKFpSPAER |

Table S3. **Primer pairs used for site-directed mutagenesis**

| Primer | Sequence (5′–3′) |
|---|---|
| S1016A-forward | AAGCGCTGCTGAGAAGAGGAAAGCCAAATG |
| S1016A-reverse | CAGCAGCGCTTAAAGGAAGCGCTTTCCCC |
| S1016D-forward | AAGCGATGCTGAGAAGAGGAAAGCCAAATG |
| S1016D-reverse | CAGCATCGCTTAAAGGAAGCGCTTTCCCC |
| S1016E-forward | AAGCGAGGCTGAGAAGAGGAAAGCCAAATG |
| S1016E-reverse | CAGCCTCGCTTAAAGGAAGCGCTTTCCCC |
| S1728A-forward | GCACGCACCGGGGGTCCTGACAGACCTGCGG |
| S1728A-reverse | CCGGTGCGTGCTGCCGCGGGTCTTCATAAAG |
| S1852A-forward | CCGTGCACCTGTGCGAGAATTGCTTGAAATG |
| S1852A-reverse | CAGGTGCACGGGGTACATTTGCAGAAAACTT |

Table S4. **Primer pairs used for ChIP and quantitative RT-PCR analysis**

| Primer | Sequence (5′–3′) |
|---|---|
| GAPDH-fwd (chromosome 12) | AACCTGCCAAATATGATGAC |
| GAPDH-rev (chromosome 12) | AGGAAATGAGCTTGACAAAG |
| DS2-fwd (chromosome 6) | TGCCGGTCTCCTAGAAGTTG |
| DS2-rev (chromosome 6) | GCGCTTGATTTCCCTGAGT |
| DS1-fwd (chromosome 1) | GATTGGCTATGGGTGTGGAC |
| DS1-rev (chromosome 1) | CATCCTTGCAAACCAGTCCT |
| 300-fwd (chromosome 1) | AGGACTGGTTTGCAAGGATG |
| 300-rev (chromosome 1) | ACCCCCATCTCAAATGACAA |
| 500-fwd (chromosome 1) | CCTGGATATGAGTTTGATCAGC |
| 500-rev (chromosome 1) | CTCTCCTTTCGCTGACACTG |
| 1000-fwd (chromosome 1) | AGGAATTGACTGCGGTGTTC |
| 1000-rev (chromosome 1) | GGGGAGGAGGAAAGGTGTAG |
| 2000-fwd (chromosome 1) | GCCATAACAGAGGGTGGAAA |
| 2000-rev (chromosome 1) | AACTTTAGGATGGGGCTGCT |

fwd, forward; rev, reverse.
